# Repopulation of T, B, and NK cells following alemtuzumab treatment in relapsing-remitting multiple sclerosis

**DOI:** 10.1186/s12974-020-01847-9

**Published:** 2020-06-15

**Authors:** Wendy Gilmore, Brett T. Lund, Peili Li, Alex M. Levy, Eve E. Kelland, Omid Akbari, Susan Groshen, Steven Yong Cen, Daniel Pelletier, Leslie P. Weiner, Adil Javed, Jeffrey E. Dunn, Anthony L. Traboulsee

**Affiliations:** 1 Department of Neurology, Keck School of Medicine, University of Southern California, 1333 San Pablo Street McKibben Hall Room 245A, Los Angeles, CA 90033 USA; 2 Department of Molecular Microbiology and Immunology, Keck School of Medicine, University of Southern California, Los Angeles, CA USA; 3 Department of Preventive Medicine, University of Southern California, Los Angeles, CA USA; 4 Department of Radiology, University of Southern California, Los Angeles, CA USA; 5 Department of Neurology, University of Chicago School of Medicine, Chicago, IL USA; 6 Department of Neurology, Stanford University, Palo Alto, CA USA; 7 Department of Neurology, University of British Columbia, Vancouver, CA USA

**Keywords:** Multiple sclerosis, Alemtuzumab, Lymphopenia, Lymphocyte repopulation, Immune regulation, Tolerance, Drug mechanisms, T cell subsets, B cell subsets, Natural killer cell subsets

## Abstract

**Objective:**

To characterize long-term repopulation of peripheral immune cells following alemtuzumab-induced lymphopenia in relapsing-remitting MS (RRMS), with a focus on regulatory cell types, and to explore associations with clinical outcome measures.

**Methods:**

The project was designed as a multicenter add-on longitudinal mechanistic study for RRMS patients enrolled in CARE-MS II, CARE-MS II extension at the University of Southern California and Stanford University, and an investigator-initiated study conducted at the Universities of British Columbia and Chicago. Methods involved collection of blood at baseline, prior to alemtuzumab administration, and at months 5, 11, 17, 23, 36, and 48 post-treatment. T cell, B cell, and natural killer (NK) cell subsets, chemokine receptor expression in T cells, in vitro cytokine secretion patterns, and regulatory T cell (Treg) function were assessed. Clinical outcomes, including expanded disability status score (EDSS), relapses, conventional magnetic resonance imaging (MRI) measures, and incidents of secondary autoimmunity were tracked.

**Results:**

Variable shifts in lymphocyte populations occurred over time in favor of CD4+ T cells, B cells, and NK cells with surface phenotypes characteristic of regulatory subsets, accompanied by reduced ratios of effector to regulatory cell types. Evidence of increased Treg competence was observed after each treatment course. CD4+ and CD8+ T cells that express CXCR3 and CCR5 and CD8+ T cells that express CDR3 and CCR4 were also enriched after treatment, indicating heightened trafficking potential in activated T cells. Patterns of repopulation were not associated with measures of clinical efficacy or secondary autoimmunity, but exploratory analyses using a random generalized estimating equation (GEE) Poisson model provide preliminary evidence of associations between pro-inflammatory cell types and increased risk for gadolinium (Gd+) enhancing lesions, while regulatory subsets were associated with reduced risk. In addition, the risk for T2 lesions correlated with increases in CD3+CD8+CXCR3+ cells.

**Conclusions:**

Lymphocyte repopulation after alemtuzumab treatment favors regulatory subsets in the T cell, B cell, and NK cell compartments. Clinical efficacy may reflect the sum of interactions among them, leading to control of potentially pathogenic effector cell types. Several immune measures were identified as possible biomarkers of lesion activity. Future studies are necessary to more precisely define regulatory and effector subsets and their contributions to clinical efficacy and risk for secondary autoimmunity in alemtuzumab-treated patients, and to reveal new insights into mechanisms of immunopathogenesis in MS.

**Trial registration:**

Parent trials for this study are registered with ClinicalTrials.gov: CARE-MS II: NCT00548405, CARE-MS II extension: NCT00930553 and ISS: NCT01307332.

## Background

Currently approved disease modifying drugs for patients with relapsing-remitting multiple sclerosis (RRMS), an autoimmune demyelinating disease of the CNS, target cells of the immune system to achieve clinical efficacy [1, 2]. Most have been reported to act in variable fashion to suppress or block pro-inflammatory CD4+ T cell types and cytokines, such as IFN-γ-secreting Th1 cells and IL-17A-secreting Th17 CD4+ T cells, and to promote immune regulation via effects on classical foxP3+ Tregs, Th2 cells, and IL-10 secreting cells. MS drugs may also target CD8+ T cells [3, 4], natural killer (NK) cells [5], antigen presenting cells [6], and more recently, B cells [7–9]. In addition, fingolimod [10], cladribine [11], glatiramer acetate [12], and others (reviewed by Longbrake and colleagues [13]) may differentially affect B cell subsets, especially memory B cells [14]. Characterization of persistent changes in immune cell types and functions in response to treatment has potential to advance our understanding of disease pathogenesis and to promote development of more effective and refined treatment strategies. Furthermore, long-term analyses of immune phenotypes that prevail or change over time could be crucial to the assessment of the risk of future disease activity.

Alemtuzumab (Lemtrada, Genzyme, Cambridge, MA) is a humanized monoclonal antibody against CD52, a glycoprotein expressed on the surface of most lymphoid, and to a lesser extent, myeloid cell types. It was approved in the USA for the treatment of RRMS in 2014, based on durable clinical efficacy, in spite of carrying a high risk for development of secondary autoimmunity [15–19]. The standard treatment protocol consists of two annual courses of i.v. alemtuzumab (12 mg/day) for five consecutive days at baseline and three consecutive days 12 months later (M12), with provisions for additional 3-day courses as needed.

Alemtuzumab induces rapid and profound lymphopenia within days of infusion, accompanied by more subtle and transient effects on monocytes, NK cells, dendritic cells, and neutrophils [16, 20–22]. Lymphopenia is followed by repopulation of B cells, CD8+ T cells, and CD4+ T cells in sequence over time, with persistent depletion of CD4+ T cells for 3 to 4 years [23–25]. The temporal pattern of repopulation, accompanied by persistent redistribution of T cell subsets in favor of circulating memory and regulatory T cells and against pro-inflammatory cytokines and Th1 and Th17 subsets, are considered to be fundamental to mechanisms of action that lead to durable efficacy in the absence of ongoing treatment [24, 26–30]. However, changes in B cells [22, 26, 31] and innate-like lymphoid cells [21], including NK cells, have also been observed, suggesting that efficacy and the development of secondary autoimmunity involve greater mechanistic complexity.

The goals of this multicenter cohort alemtuzumab treatment study were as follows: (1) to analyze the repopulation of immune cells over time, with a focus on the balance between regulatory T cell, B cell, and NK cell subsets and effector subsets in each compartment; (2) to assess Treg functional competence over time; (3) to identify changes in T cells that express chemokine receptors; (4) and to explore associations of immune measures with disease activity and the risk of secondary autoimmunity.

## Methods

### Study design

This project was designed as a prospective add-on mechanistic multicenter study for patients enrolled in CARE-MS II, CARE-MS II extension (CARE-MS II Ext) at the University of Southern California (USC) and Stanford University, and an open label investigator-sponsored study (ISS) conducted at the Universities of British Columbia (UBC) and Chicago (UC). Thirty-seven patients were initially enrolled, eight were excluded due to baseline (M0) sample shipping delays; final enrollment was 29 patients. The first patient was enrolled in January of 2009, the final patient was enrolled in June of 2013, and the last blood sample was collected in May of 2016. Patients received two annual courses of i.v. alemtuzumab (12 mg/day) for 5 days at baseline (M0) and for 3 days 12 months later (M12), except for one CARE-MS II patient, who was randomized to receive 24 mg/day; 11 patients received a third course on study or shortly thereafter. Inclusion and exclusion criteria for CARE-MS II [15, 16] and ISS have been described previously [32]. Demographics and baseline characteristics of the patients are illustrated in Table 1.

**Table 1:** Patient demographic and baseline characteristics

Characteristic	Values
Number enrolled	29
Sex: female/male	21/8 (74% female)
Age at baseline: mean ± SD years	33.9 ± 8.9
Ethnicity (self-reported): number (%)	
White (non-Hispanic)	14 (48.2%)
Hispanic/Latino/Mexican	4 (13.8%)
African American	6 (20.7%)
Asian	4 (13.8%)
Unknown	1 (3.45%)
Treatment naïve at baseline: number (%)	6 (20.7%)
Disease duration from diagnosis: mean ± SD years	3.1 ± 2.5
Disease duration from symptom onset: mean ± SD years	4.7 ± 3.8
Number of relapses 2 years prior to baseline: mean ± SD	2.5 ± 0.7
Baseline EDSS: mean ± SD	3.1 ± 1.1

### Clinical assessments

Clinical, conventional imaging, and safety assessments were conducted as described for the original CARE-MS studies (ClinicalTrials.gov identifiers NCT00548405 and NCT00930553 and [16], modified as described by Vavasour and colleagues [33]. Data were available for up to 28 patients. EDSS was assessed at 6-month intervals for 2 years, and for a small number of patients (*N* = 6) at M36 and M48. The number of new T2 or Gd+ lesions were identified by comparison with prior scans, where applicable, on an annual basis for 4 years, accompanied by assessment of changes in total T2 lesion volume and brain parenchymal fraction (BPF). Relapses, defined as new MS symptoms lasting at least 48 h and confirmed by neurological examination, were recorded. The incidence and timing of new symptomatic secondary autoimmunity were tracked according to trial safety monitoring requirements, which included blood testing for evidence of autoimmune thyroid disease, immune thrombocytopenia (ITP), and autoimmune nephropathy.

### Blood collection and shipment

Venous blood was collected at M0, prior to alemtuzumab infusion, and at M5, M11, M17, M23, M36, and M48 from patients at UBC, UC, and Stanford and shipped overnight at ambient temperature to USC for processing and assay performance. To maintain as much consistency as possible in the time from blood collection to processing in the laboratory, samples arriving at USC after 12:00 noon PST were excluded from further study. Samples collected at USC were stored overnight at room temperature and processed the next day.

### Lymphocyte phenotype analyses by flow cytometry

Three strategies were used to assess changes in percentages of lymphocyte phenotypes using 4-color fluorescence-activated cells sorting (FACS). First, a general survey was conducted in whole blood using standard staining procedures as described previously [34] and fluorochrome-labeled antibodies specific for CD3+ T cells (CD3-APC, clone UCHT1), CD3+CD4+ T cells (CD4-PE and PECy5, clone RPAT4), CD3+CD8+ T cells (CD8-FITC, clone RPAT8), CD3-CD19+ B cells (CD19-FITC, clone HIB19), CD3-CD56+ NK cells (CD56-PE, clone B159), CD3+CD56+ NKT cells, CD3+CD4+ T cells that express CD45RA (total naïve; CD45RA-FITC, clone HI100), or CD45RO (total memory; CD45RO-FITC, CD45RO-PE, clone UCHL1). To evaluate changes in naïve and memory T cell subsets in the CD4+ T cell compartment, the whole blood FACS panel also included antibodies against CD27 (CD27-APC, clone M-T271 or O323) to identify CD4+CD27+CD45RA naïve T cells, CD4+CD27+CD45RA- central memory T cells (TCM), CD4+CD27-CD45RA- effector memory T cells (TEM), and CD4+CD27-CD45RA+ new effector T cells (new Teff), according to Lovett-Racke et al. [35]. Antibodies against VLA-4 (very late antigen 4, VLA-4-PE, clone 9F10) and chemokine receptors CXCR3, CCR5, CCR3, and CCR4 were included to assess the potential for CD3+CD4+ and CD3+CD8+ T cells to migrate and enter tissue, and for the chemokine receptors, as surrogate markers of pro-inflammatory T helper 1 (Th1: CXCR3 and CCR5) and anti-inflammatory T helper 2 (Th2: CCR3 and CCR4) cells. Antibodies were purchased from BD Biosciences, San Jose, CA, and eBioscience/ThermoFisher Scientific, Grand Island, NY.

Second, changes in Tregs were assessed in PBMC isolated from heparinized blood by density gradient centrifugation using Ficoll-Paque-Plus (GE Healthcare, 17-1440-02; Sigma Aldrich, St. Louis, MO), as described previously [36]. When possible, excess PBMCs were stored in liquid nitrogen for future studies. Identification of classical Tregs, defined as CD4+CD25^hi^CD127^lo/neg^ foxP3+ cells was accomplished using kits purchased from eBioscience/ThermoFisher according to the manufacturer’s instruction and similar to methods described by Jones and colleagues [37] and consistent with minimal requirements recommended for Treg identification [38]. Effector T cells (Teff) were defined as CD4+CD25+CD127+ cells lacking expression of foxP3. The panel also included anti-CD39, CD45RA, and CD45RO and consisted of CD4-PerCP-Cy5.5 (clone RPAT4), CD127-FITC (clone EBioRDR5), CD45RA-FITC (clone HI100), CD45RO-PE (clone UCHL1), CD25-PE (clone MA251), CD39-FITC (clone eBioA1), and foxP3-AlexaFluor 647 (clone 150D). Antibodies were purchased from BD Biosciences or eBioscience/ThermoFisher Scientific with the exception of foxP3, which was from BioLegend (San Diego, CA).

Third, changes in CD19+CD20+ B cell subsets were assessed in PBMC thawed from storage in liquid nitrogen. The FACS panel included antibodies against CD19, CD20, CD38, CD27, and CD24 to allow identification of total memory B cells as CD19+CD20+CD27+, total naïve B cells as CD19+ CD20+ CD27-, and “transitional” or naïve “regulatory” B cells as CD19+ CD20+ CD27-CD24^hi^CD38^hi^, similar to that described by Kim et al. [39]. Antibodies were as follows: CD19-FITC (clone HIB19), CD20-FITC (clone 2H7), CD38-PerCP (clone HIT2), CD27-APC (clone M-T271), and CD24-PE (clone ML5), purchased from BD Biosciences or BioLegend.

Staining conditions and FACS settings, including photomultiplier tube (PMT) voltages and compensations were defined in preliminary experiments and used throughout the study. Data acquisition was accomplished using a FACSCalibur flow cytometer (BD Biosciences, San Jose, CA) and analyses accomplished using the FlowJo software (version 7.6.5, Ashland, OR). Gating strategies for stained populations were applied in combination with staining by appropriate isotype control antibodies for all assays.

Cell counts for primary lymphocyte populations in a subset of patients were also available from clinical phenotyping (TBNK panel) or complete blood counts (CBC, with differential) performed every 6 months (M0, M6, M12, M18, M24, M36, and M48) by Quest or Covance Laboratories.

### Functional regulatory T cell assay

**Fig. 1:**
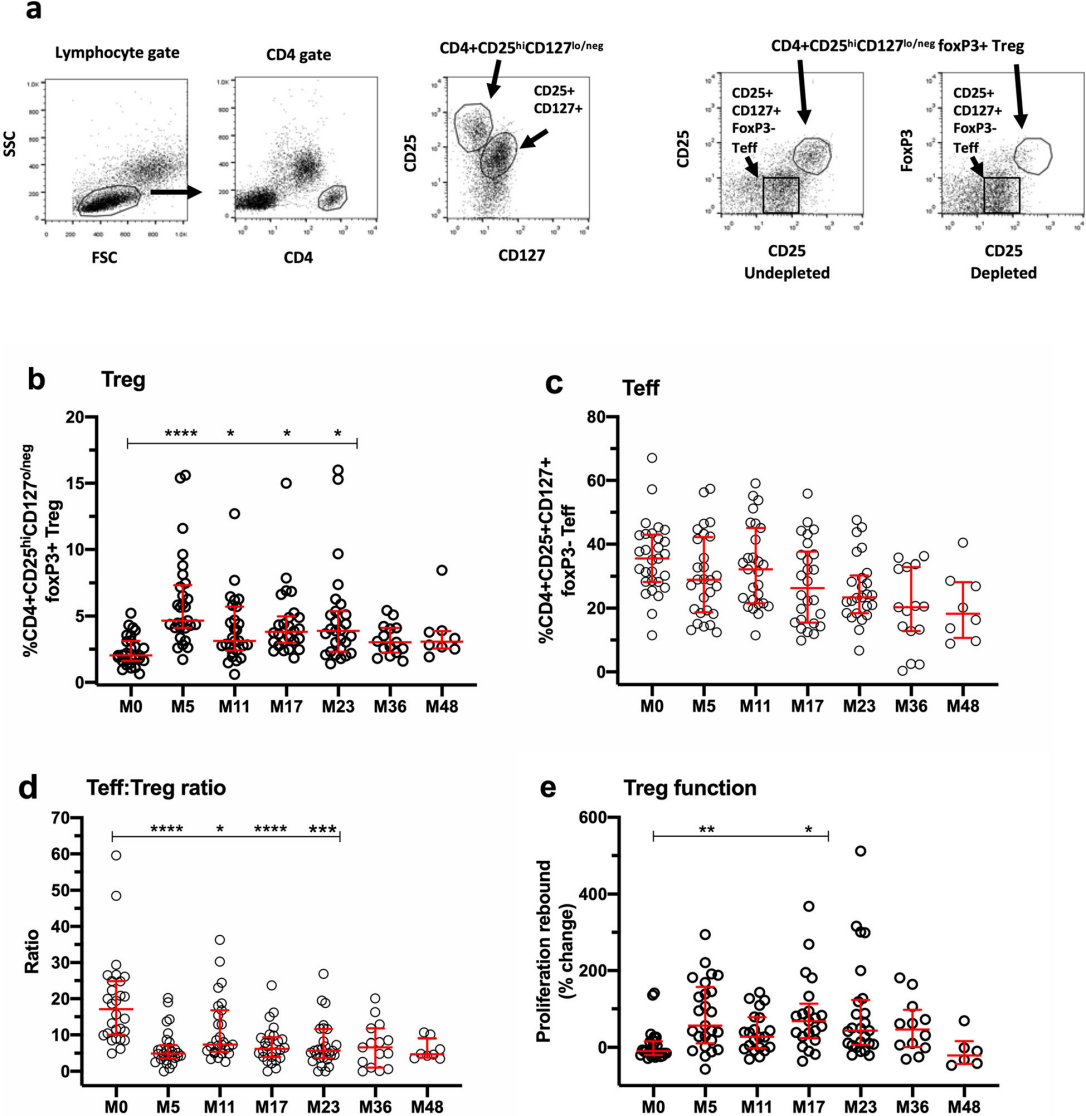
Changes in CD4+ regulatory T cells (Treg), “effector” T cells (Teff), and Treg function in PBMC. **a** FACS gating strategy used to identify CD4 + CD25^hi^CD127^lo/neg^ foxP3+ Tregs and CD4 + CD25 + CD127 + foxP3- Teff in PBMC (first four FACS plots, left to right) and in PBMC depleted of CD25+ Tregs (fifth FACS plot, right). **b-e** Illustrate changes over time at each study timepoint for each individual patient (open symbols), compared with baseline (M0) values. **b** Changes in Tregs. **c** Changes in Teff. **d** Changes in Teff:Treg ratio. **e** Changes in Treg function, indicated as percent rebound in CD25-depleted PBMC compared to undepleted PBMC. Data in all panels represent median/IQR values for all patients at each timepoint. *p* values indicate significant changes from baseline (M0) as follows: ****p ≤ 0.0001, ***p ≤ 0.001, **p ≤ 0.01, *p ≤ 0.05; mixed effects ANOVA with Tukey’s corrections for multiple comparisons

Treg function is typically assessed by the ability of purified CD4+CD25^hi^ suppressor cells to inhibit proliferation or cytokine secretion by mitogen- or antigen-activated CD4+CD25- target cells in vitro [40, 41]. These assays require freshly isolated cells in a number sufficient for purification, presenting a practical challenge for lymphopenic patients and in samples shipped overnight. For this reason, an alternative assay was developed in which the presence of functional Tregs is revealed by rebound proliferation in PBMC depleted of CD25+ cells (CD25-depleted PBMC), compared with unfractionated PBMC containing CD25+ cells (CD25-undepleted PBMC). This approach is justified by the fact that strategies to deplete CD25+ T cells formed the original basis for identification of Tregs in mouse models of autoimmune disease [42] and by its use to detect Tregs in PBMC from systemic lupus erythematosus patients [43]. Depletion was accomplished using anti-CD25-coupled magnetic microbeads and magnetic cell separation columns (MS columns) available from Miltenyi Biotec (human CD25 Microbeads II, Auburn, CA), according to the manufacturer’s protocol. Success of depletion was confirmed in all samples by FACS (see Fig. 1a) employing the same antibodies used for Treg identification, with 88–95% reduction of CD4+CD25^hi^ cells, and 81–95% depletion in cells stained for CD4, CD25^hi^, and foxP3 (data not shown). Rebound proliferation was measured in PBMC and CD25-depleted PBMC plated separately at 2 × 10^5^ cells/well and stimulated with 5 μg/mL PHA for 4 days. Tritiated thymidine (3H TdR, 1 μCi/well; MP Biomedicals) was added for the last 18–24 h. Cells were harvested onto glass fiber filter strips and counts per minute (cpm) ^3^H-TdR measured in a liquid scintillation counter. Data are expressed as percent increase in mean cpm in CD25-depleted PBMC compared with undepleted PBMC.

### Analyses of cytokine secretion patterns and percentages of Th1 and Th17 cells in PBMC

Cytometric bead array (CBA) kits from BD Pharmingen (San Diego, CA) were used to measure concentrations of IL-2, IL-4, IL-6, IL-7, IL-8, IL-9, IL-10, IL-12p70, IL-13, IL-17A, IL-21, MCP1, RANTES, fractalkine, TNF-α, IFN-γ, and GM-CSF in 48-h supernatants from PBMC plated in triplicate at 2 × 10^5^ cells/well in 96 well plates (Corning Costar) in the presence and absence of phytohemagglutinin (PHA; 5ug/mL). Supernatants were stored at − 20 °C until used for assays, when they were thawed and incubated with appropriate bead fluorochrome pairs in triplicate, according to the manufacturer’s protocol and as reported previously [44]. Data were acquired using a BD Accuri C6 flow cytometer (BD Biosciences, San Jose, CA) and analyzed using CBA FCAP Array software, v3.0 (BD Biosciences). Concentrations were extrapolated from standard curves included for each analyte. Assays were performed in batches that included all timepoints for each individual patient. Inter-assay variability was assessed by inclusion of standard supernatants prepared from a bulk culture of PBMC collected from a control subject and stimulated with 5 μg/mL PHA, and by spiking the assays with known concentrations of each cytokine. Inter-assay variability was less than 10% (data not shown). Samples were available from M0, M5, M11, M17, and M23 timepoints.

Changes in Th1 and Th17 cells in PBMC were assessed using procedures adapted from intracellular cytokine staining and leukocyte activation cocktail kits available from BD Biosciences (San Jose, CA). Briefly, 1-2 × 10^6^ PBMC/mL were suspended in culture medium and placed at 37 °C, 5% CO_2_ for 2 h prior to the addition of leukocyte activation cocktail (phorbol 12-myristate 13-acetate, or PMA and ionomycin at 50 ng/mL and 500 ng/mL, respectively) in the presence of the protein transport inhibitor, brefeldin A at 10 μg/mL. Culture medium consisted of RPMI 1640 (JRH Bioscience/Millipore Sigma, St. Louis, MO) supplemented with 5% heat-inactivated fetal calf serum (Omega Scientific, Tarzana, CA), 20 mM HEPES, 0.1 mM non-essential amino acids, 4 mM L-glutamine, 1 mM sodium pyruvate, 100 units/mL penicillin/streptomycin, and 50 μM 2-mercaptoethanol. PBMC cultured in the absence of leukocyte activation cocktail served as controls. After overnight incubation, PBMC were stained for surface CD4 expression followed by fixation/permeabilization and staining to detect intracellular IFN-γ (Th1; IFN-γ-FITC, clone 25,723.11) or IL-17A (Th17; IL-17A-PE, clone eBio65DEC17). Data were acquired on a FACSCalibur flow cytometer and analyses conducted using the FlowJo software. The gating strategy involved identification of lymphocytes in FSC/SSC plots and gating on CD4+ lymphocytes to identify CD4+IFN-γ+and CD4 + IL-17A+ cells. Analyses also included IFN-γ+ and IL-17A+ cells in the CD4- gate. Antibodies were purchased from BD Biosciences or eBioscience/ThermoFisher Scientific. Staining conditions and FACS settings were defined in preliminary experiments and used throughout the study.

### Statistical analyses

The study was originally powered to detect a minimum 45% change in the percentage of Tregs at 70% variability at any timepoint from M5, M11, M17, and M23 compared with baseline, to achieve a power of 0.8 at *p* ≤ 0.05 (paired *t* test). Based on these assumptions, 21 patients were required; 29 were enrolled to achieve this goal and 22 completed all M0-M23 timepoints. Subsequently, the study was amended to add M36 and M48 timepoints; 15 patients completed all timepoints from M0-M36, and 5 completed all M0-M48 timepoints. The maximum number of samples available for assays at each timepoint was 29 (M0, M5), 27 (M11), 26 (M17, M23), 15 (M36), and 8 (M48) for most measures in whole blood and PBMC, depending on cell yields. For thawed PBMC used to identify B cell subsets, the maximum number of samples available for each timepoint was 23 (M0), 10 (M5), 14 (M11), 5 (M17), 16 (M23), 11 (M36), and 4 (M48). For clinical TBNK analyses, samples were available for up to 20 patients at M0, M6, M12, M18, M24, M36, and M48. Statistical significance of changes in lymphocyte measures and in BPF and T2 volume at each timepoint compared with baseline was assessed using a mixed effects analysis of variance (ANOVA) model with Tukey’s corrections for multiple comparisons. Exceptions occurred for analyses of changes in B cell subsets, cytokine concentrations in PBMC supernatants, EDSS, and number of new T2 and Gd+ lesions, in which Kruskal-Wallis test for non-parametric data was used. Graphpad Prism, v8 was used for these analyses; *p* values ≤ 0.05 are considered statistically significant.

Post hoc analyses of correlations between longitudinal changes in lymphocyte, clinical, and imaging parameters, between- and within-individuals were conducted on data from patients enrolled at USC, UBC, and UC and employed a linear mixed effects model for repeated measures described by Irimata et al. [22], using a SAS macro (SAS 9.4). Associations between percentages of specific lymphocyte subsets and EDSS, BPF, number of new T2 lesions, T2 lesion size, and number of new Gd+ lesions were assessed. In addition, immunological data were stratified for patients with active and stable disease, and for patients who did or did not develop secondary autoimmunity during the 4-year study period. Active disease was defined as the presence of relapses and/or new T2 lesions any time on study. Stable disease was defined as the absence of relapses and the absence of new T2 lesions on study.

An additional exploratory analysis strategy involved the use of a random generalized estimating equation (GEE) Poisson distribution model to determine if immune measures change in association with risk of new Gd+ or T2 lesions (SAS 9.4). Briefly, GEE adopts the nested structure of within- and between-individual data and allows application of a random effect to estimate within-individual risk. The dispersion parameter ϕ was estimated as the ratio of deviance, with correction if overdispersion was detected. This method standardizes immune measures that vary in scale to a mean z score of 0 and standard deviation of 1. This allows comparisons and ranking of the strength of associations across all measurements, such that a rate ratio (rr) represents increased relative risk associated with one standard deviation increase in immune measures. A Benjamini-Hochberg procedure was used to adjust error for multiple comparisons and reduce false discovery rates. *p* values ≤ 0.05 are considered statistically significant.

## Results

### Changes in Tregs and Teff in PBMC over time

Figure 1a illustrates the FACS gating strategy used to identify classical CD4+CD25^hi^CD127^lo/neg^ foxP3+ Tregs and CD4+CD25+CD127+foxp3- Teff in PBMC. Briefly, lymphocytes were identified in forward scatter/side scatter plots (FSC/SSC; lymphocyte gate) followed by gating on CD4+ lymphocytes (CD4 gate) to identify CD4+CD25^hi^CD127^lo/neg^ and CD4+CD25+CD127+ cells (see middle plot), with CD25 (clone M-A251) on the *y*-axis and CD127 on the *x*-axis. The percentage of classical Tregs increased following alemtuzumab treatment, most clearly evident at the earliest timepoint (M5), followed by more modest, but significant elevation until M23 (Fig. 1b). By contrast, the proportion of CD4+CD25+CD127+foxP3- Teff cells showed a non-statistically significant trend for reduction over the study period (Fig. 1c). Overall, ratios of Teff:Treg were significantly reduced from M5 to M23 (Fig. 1d).

The majority of Tregs were CD39+ (Supplementary Figure S2A & D), with CD39 expression occurring in both foxP3+ (Figure S2A & B) and foxp3- T cells (Figure S2C). CD39+ cells were also found on CD4+ T cells that did not express CD25 (Figure S2E). Tregs exhibited a predominantly CD45RO+ memory phenotype, increasing significantly at M5 and M23 (Figure S2F), while the percentage of CD45RA+ Tregs was significantly reduced at M5 (Figure S2G).

### Functional capacity of Tregs

Treg function was assessed by measuring the presence of rebound proliferation in PBMC depleted of CD25+ cells, as described in the methods. Results are presented in Fig. 1e. Significant rebound in PHA-induced proliferation was observed at M5 and M17 (*p* = 0.01 and 0.05, respectively), comparing PBMC with CD25-depleted PBMC. This suggests not only that Tregs are functional, but that they may exhibit enhanced function in the early phase of repopulation after each course of alemtuzumab treatment.

### Changes in patterns of repopulation by T cells expressing chemokine receptors

**Fig. 2:**
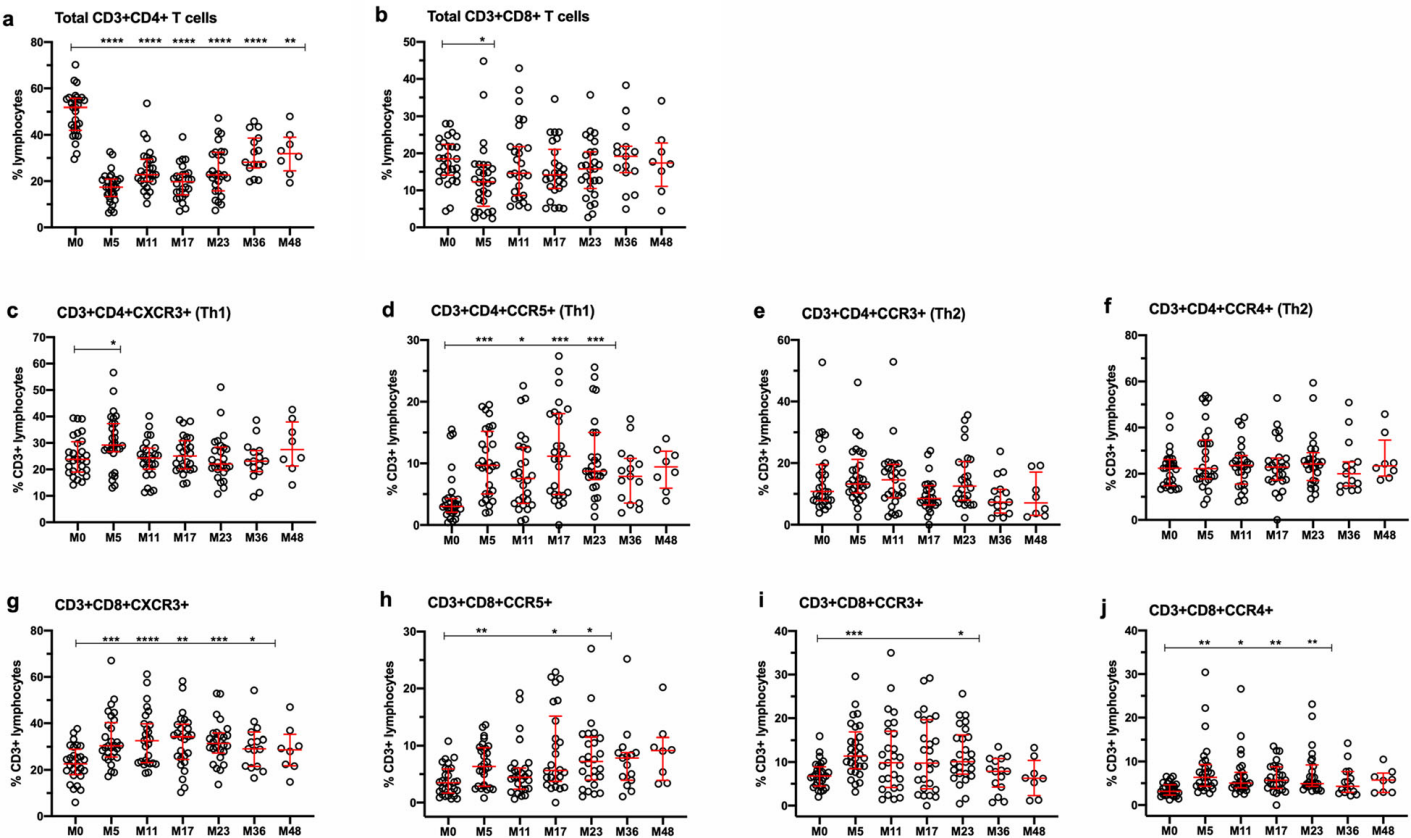
Changes in CD4+ and CD8+ T cells expressing chemokine receptors in whole blood. **a** Total CD3+CD4+ T lymphocytes. **b** Total CD3+ CD8+ T cells. **c** and **d** Show CD3+CD4+ T cells expressing CXCR3 and CCR5, respectively, which are enriched in Th1 cells. **e** and **f** Show CD3+CD4+ T cells expressing CCR3 and CCR4, respectively, which are enriched in Th2 cells. **g**–**j** Show CD3+CD8+ T cells that express CXCR3, CCR5, CCR3, and CCR4, respectively. Data represent median/IQR values for all patients at each timepoint; open circles indicate data points for each individual patient. *p* values indicate significant changes from baseline (M0) values as follows: ****p ≤ 0.0001, ***p ≤ 0.001, **p ≤ 0.01, *p ≤ 0.05; mixed effects ANOVA with Tukey’s corrections for multiple comparisons

Chemokine receptors have been reported to be differentially expressed in Th1 (CXCR3 and CCR5) and Th2 cells (CCR3 and CCR4) [45–48], and CXCR3 is also upregulated in Th1 and CD8+ effector T cells upon activation [49, 50]. Thus, it was of interest to evaluate chemokine receptor expression in T cells during repopulation. Figure 2a and b, respectively, shows changes in the percentage of total CD3+CD4+ and CD3+CD8+ T cells in whole blood. The percentage of CD3+CD4+ T cells expressing CXCR3 (Fig. 2c) or CCR5 (Fig. 2d), presumed Th1-like cells, increased significantly at M5, with sustained elevation of CD3+CD4+CCR5+ T cells until M23, compared with M0. The percentage of CD3+CD4+ T cells expressing CCR3 or CCR4, enriched in Th2 cells, did not vary significantly over time (Fig. 2e and f, respectively). For CD3+CD8+ T cells, the proportion of CXCR3 (Fig. 2g), CCR4+ (Fig. 2j), and to a lesser extent, CCR5+ (Fig. 2h) and CCR3+ (Fig. 2i) cells increased significantly from M5 to M36. The majority (greater than 90%) of CD3+CD4+ and CD3+CD8+ T cells expressed VLA-4 with no change over time (data not shown).

### Changes in naïve and memory CD4+ T cell subsets in whole blood

It has been well-documented that total CD4+CD45RA+ naïve T cells are reduced, and total CD4+CD45RO+ memory T cells are spared following alemtuzumab treatment [23, 30]. Our data show similar changes in total memory (supplementary Figure S3A) and total naïve CD4+ T cells (Figure S3B). Figure S3 also illustrates changes over time in subsets of naïve and memory CD4+ T cells, defined as described by Lovett-Racke and colleagues [35]. Briefly, lymphocytes were identified in FSC/SSC plots, followed by gating on CD4+CD45RA+ or CD45RA- cells to identify CD27+ and CD27- lymphocytes (FACS gating strategy not illustrated). Figure S3 shows that the percentage of CD4+CD45RA+CD27+ naïve T cells (Figure S3C) was reduced at M5 and M17, while CD4+CD45RA-CD27+ central memory T cells (TCM; Figure S3D) remained relatively stable over time. The percentage of CD4+CD45RA-CD27- effector memory T cells (TEM; Figure S3E) increased at M5, along with CD4+CD45RA+ new effector T cells (new Teff; Figure S3F).

### Changes in cytokine secretion patterns in PBMC

Supplementary Figure S4 illustrates significant reductions in concentrations of IL-2 (Figure S4A) and IFN-γ (Figure S4B) from M5 to M23, while IL-17A (Figure S4C) was significantly reduced at M5, M11, and M23 compared with M0. The anti-inflammatory cytokine IL-10 showed little change except for a modest reduction at M23 (Figure S4D). Concentrations of TNF-α, IL-8, IL-6, Rantes, MCP-1, and GM-CSF were unchanged, and IL-4, IL-7, IL-9, IL-13, IL-21, and fractalkine were undetectable in PHA-activated PBMC supernatants (data not shown).

### Changes in Th1 and Th17 cells

Because Th1 and Th17 cells have been implicated in the pathogenesis of MS [2, 51], intracellular cytokine staining for IFN-γ and IL-17A was also performed in PBMC. Supplementary Figure S4 shows a significant increase in the percentage of CD4+IFN-γ+ Th1 cells (Figure S4E) only at M5 compared with M0, with no change in CD4+Th17+ cells (Figure S4F). Lymphocytes lacking CD4 expression (CD4-) were clearly capable of producing IFN-γ (Figure S4G) but showed no change in percentage throughout the study. IL-17A was not detected in CD4- cells, and CD4+ T cells capable of producing both IFN-γ and IL-17A were negligible (data not shown). IFN-γ and IL-17A were undetectable in unstimulated cells (data not shown).

### Changes in B cell subsets

**Fig. 3:**
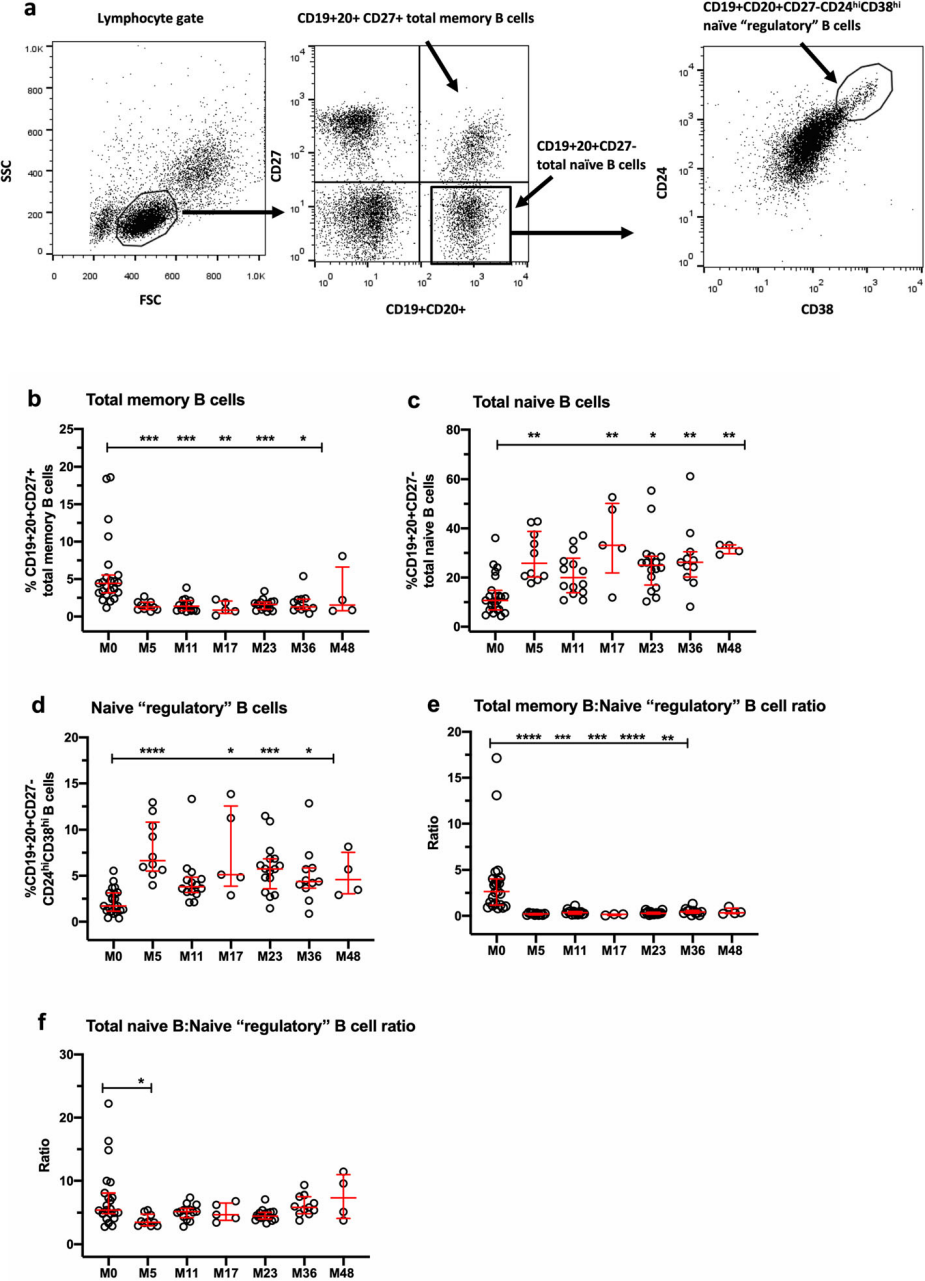
Changes in B cell subsets in thawed PBMC. **a** FACS gating strategy used to identify CD19+20+CD27+ total memory B cells, CD19+20+ total naïve B cells, and CD19+ CD20+CD27-CD24^hi^CD38^hi^ B cells. **b** Total CD19+CD20+CD27+ memory B cells. **c** CD19+CD20+CD27- total naïve B cells. **d** CD19+CD20+CD27-CD24^hi^CD38^hi^ B cells. **e** Ratio of total memory B cells to naïve “regulatory” CD24^hi^CD38^hi^ B cells. **f** Ratio of total naïve B cells to CD19+CD20+CD27-CD24^hi^CD38^hi^ B cells. Data represent median/IQR values for all patients at each timepoint; open circles indicate data points for each individual patient. *p* values indicate significant changes from baseline (M0) values as follows: ****p ≤ 0.0001, ***p ≤ 0.001, **p ≤ 0.01, *p ≤ 0.05; Kruskal-Wallis test

There is strong evidence of key roles for B cells in the pathogenesis of MS [7, 13, 14, 52–54], so evaluation of changes in B cell subsets following alemtuzumab is of considerable interest. The findings are shown in Fig. 3. Figure 3a illustrates the FACS gating strategy used to identify CD19+CD20+CD27+ memory B cells and CD19+CD20+CD27- naïve B cells in the lymphocyte gate, followed by gating on naïve B cells to identify naïve “regulatory” CD19+CD20+CD27-CD24^hi^CD38^hi^ B cells. Figure 3b shows profound, sustained depletion of the percentage of total memory B cells from M5 to M36, compared with M0, accompanied by significant elevation of total naïve B cells from M5 to M48 (Fig. 3c), indicative of hyper-repopulation. Naïve “regulatory” B cells were also significantly increased at M5, M17, M23, and M36 (Fig. 3d); cells with this phenotype have been reported to exhibit regulatory activity [10, 39]. Figure 3e indicates that the ratio of total memory B cells to naïve “regulatory” CD24^hi^CD38^hi^ B cells is markedly reduced from M5 to M36. The ratio of total CD19+CD27-naive B cells to CD19+CD27-CD24^hi^CD38^hi^ naïve “regulatory” B cells was reduced at M5 and was not sustained at later timepoints (Fig. 3f). The depletion of CD19+CD27+ memory B cells and expansion of CD19+CD27-CD24^hi^CD38^hi^ B cells were robust and occurred in most, if not all patients.

### Changes in natural killer (NK) subsets

**Fig. 4:**
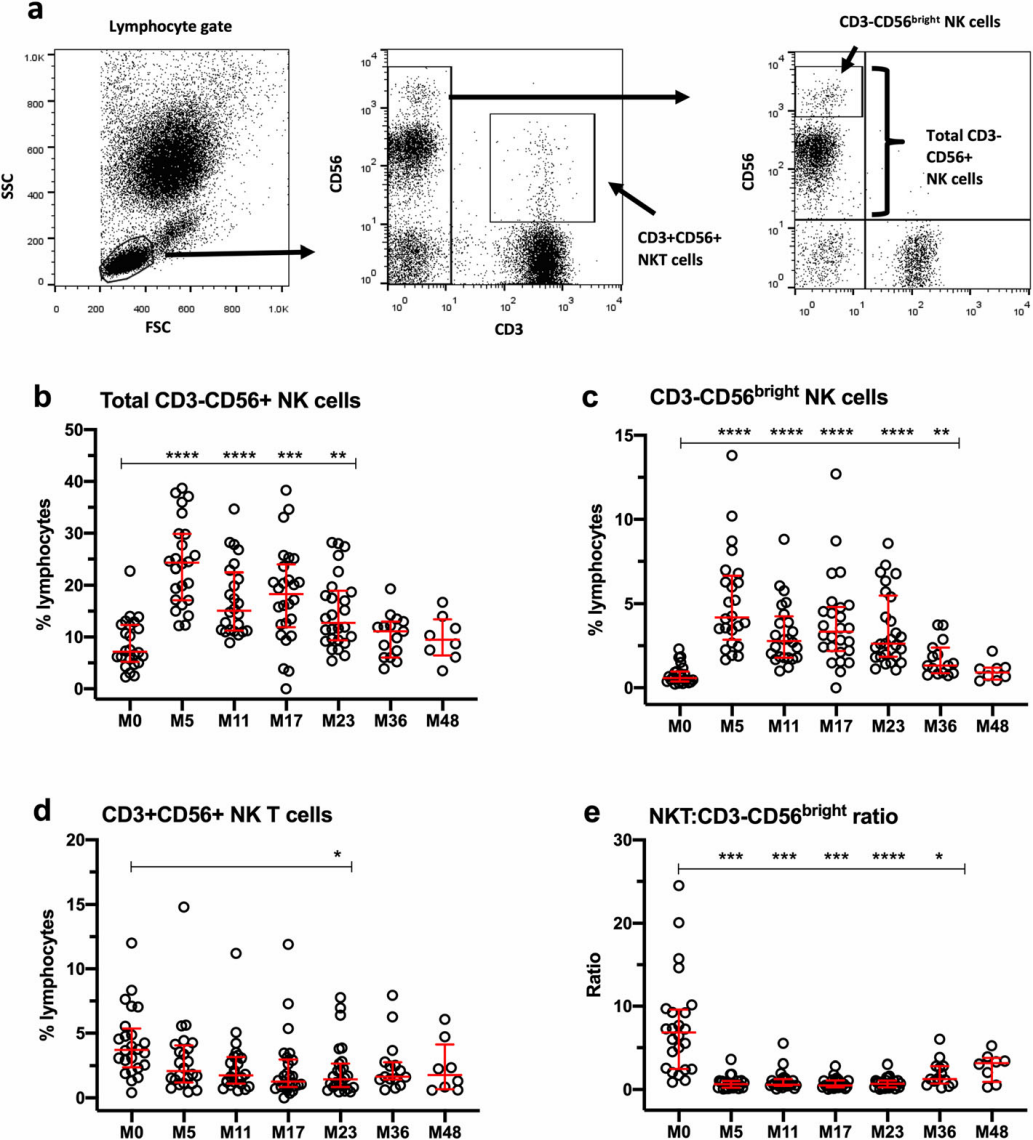
Changes in NK cell subsets in whole blood. **a** FACS gating strategy used to identify CD3-CD56+ total NK cells, CD3-CD56^bright^ NK cells, and CD3+CD56+ NKT cells. **b** Total CD3-CD56+ NK cells. **c** CD3-CD56^bright^ NK cells. **d** CD3+ CD56+ NKT cells. **e** Ratio of NKT cells:CD3-CD56^bright^ T cells. Data represent median/IQR values for all patients at each timepoint; open circles indicate data points for each individual patient. *p* values indicate significant changes from baseline (M0) values as follows: ****p ≤ 0.0001, ***p ≤ 0.001, **p ≤ 0.01, *p ≤ 0.05; mixed effects ANOVA with Tukey’s corrections for multiple comparisons

Figure 4a illustrates the FACS gating strategy used to identify total CD3-CD56+ NK cells, CD3-NK cells that express high levels of CD56 (CD3-CD56^bright^ NK cells), and CD3+CD56+ NKT cells in the lymphocyte gate, similar to methods published by Andre and Anfossi [55]. Figure 4b shows a significant increase in the percentage of total CD3-CD56+ NK cells from M5 to M23, while Fig. 4c shows a similar pattern of increase in CD3-CD56^bright^ NK cells. By contrast, the proportion of CD3+CD56+ NKT cells (Fig. 4d) remained relatively stable over time, resulting in significant reductions in the ratio of NKT cells to CD56^bright^ NK cells. (Fig. 4e).

### Clinical disease outcomes

**Fig. 5:**
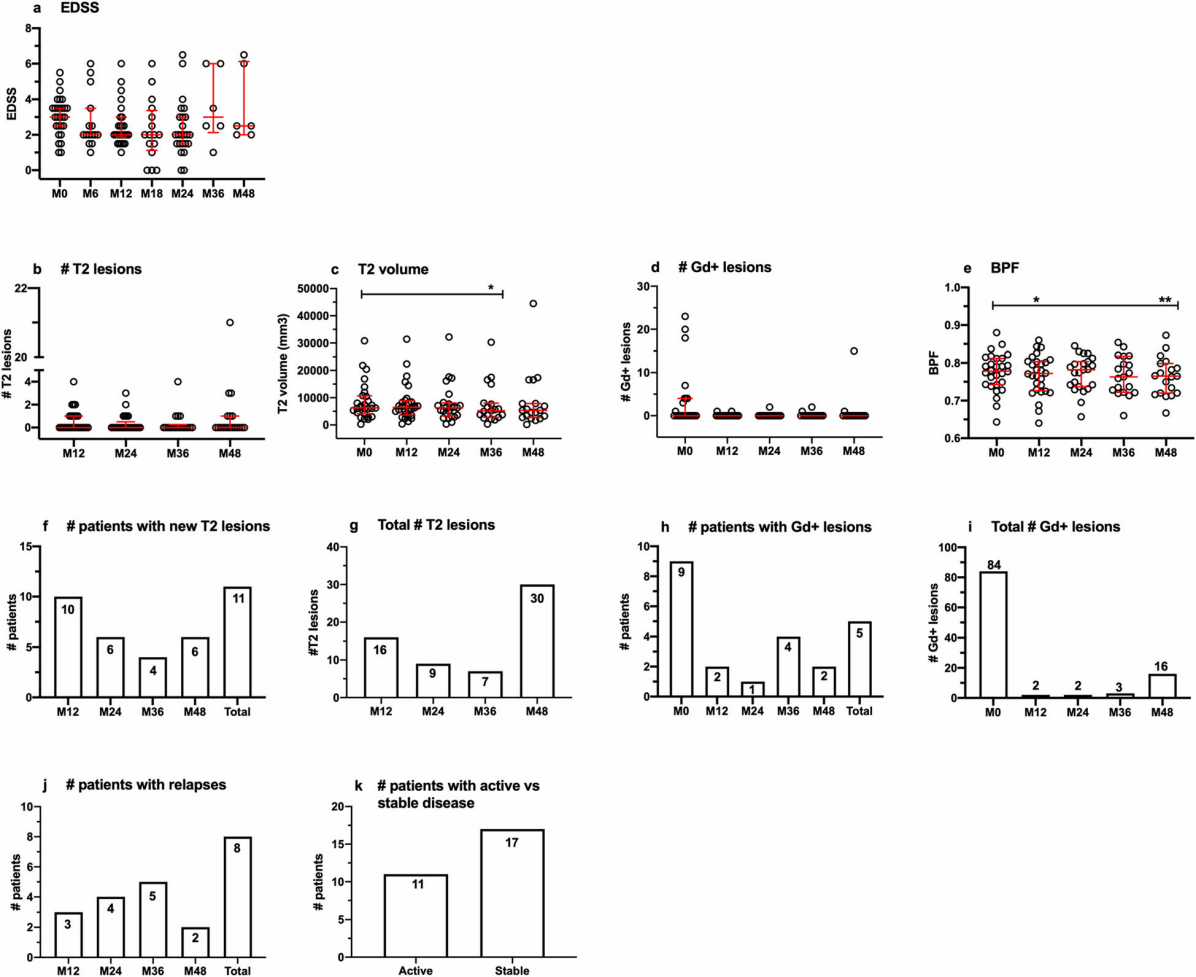
Clinical and imaging outcomes. **a** Expanded disability status scores (EDSS), measured every 6 months. **b** Number of new T2 lesions. **c** T2 volume. **d** Number of Gd+ lesions at baseline; subsequent timepoints represent new Gd+ lesions. **e** Brain parenchymal fraction (BPF). Data are expressed as median/IQR values for all patients at each timepoint. *p* values indicate significant changes from baseline (M0), where applicable, as follows: **p ≤ 0.01, *p ≤ 0.05; Kruskal-Wallis test for EDSS, number of new T2 and Gd+ lesions, and mixed effects ANOVA with Tukey’s corrections for T2 volume and BPF. **f** Number of patients with new T2 lesions in the first year (up to M12), second year (M13–M24), third year (M25–M36), and fourth year (M37–M48) accompanied by total number for all timepoints on study. **g** Total number of new lesions in each year. **h** Number of patients with Gd+ lesions at M0 [11] and new Gd+ lesions in each year, accompanied by the total number of new Gd+ lesions from M12 to M48. **i** Total number Gd+ lesions at M0 and new Gd+ lesions in each year. **j** Number of patients who experienced relapses in each year on study, accompanied by the total for all years. **k** Number of patients who experienced relapses and/or new Gd+ lesions (active disease) or had no relapses or Gd+ lesions (stable disease) on study

Patients enrolled in this study were part of a larger cohort for an individual investigator-initiated study conducted at the Universities of British Columbia and Chicago; clinical and imaging outcome data have been reported for the larger cohort [33] and are summarized for the current cohort in Fig. 5, which also included CARE-MS II and CARE-MS II extension patients enrolled at USC. Although there appeared to be a modest trend for improvements in EDSS (panel a), number of new T2 lesions (panel b) and number of new Gd+ lesions (panel d), statistically significant differences from baseline were only detectable in T2 volume (panel c) and BPF (panel e). Eleven patients developed new T2 lesions (panel f) and 5 experienced new Gd+ lesions on study (panel h). Eight patients experienced relapses (panel j), most of which occurred in the third year, for a total of 13 relapses from M0 to M48. New or secondary autoimmunity occurred in years 2 or 3, affecting eight patients (data not illustrated). Seven developed autoimmune thyroiditis and one had autoimmune thrombocytopenia. Two patients had autoimmune thyroid disease prior to baseline and were not included in the on-study count. Eleven patients were considered to have active disease, based on presence of relapses and/or new T2 lesions, while 17 patients were classified as having stable disease, based on the absence relapses and absence of new T2 lesions on study (panel k). When defined less conservatively as the presence of relapses and/or new T2 lesions and/or new Gd+ lesions, the numbers of patients with active disease vs. stable disease were 12 and 16, respectively.

### Analyses of relationships between immunological parameters and clinical disease outcomes

**Fig. 6:**
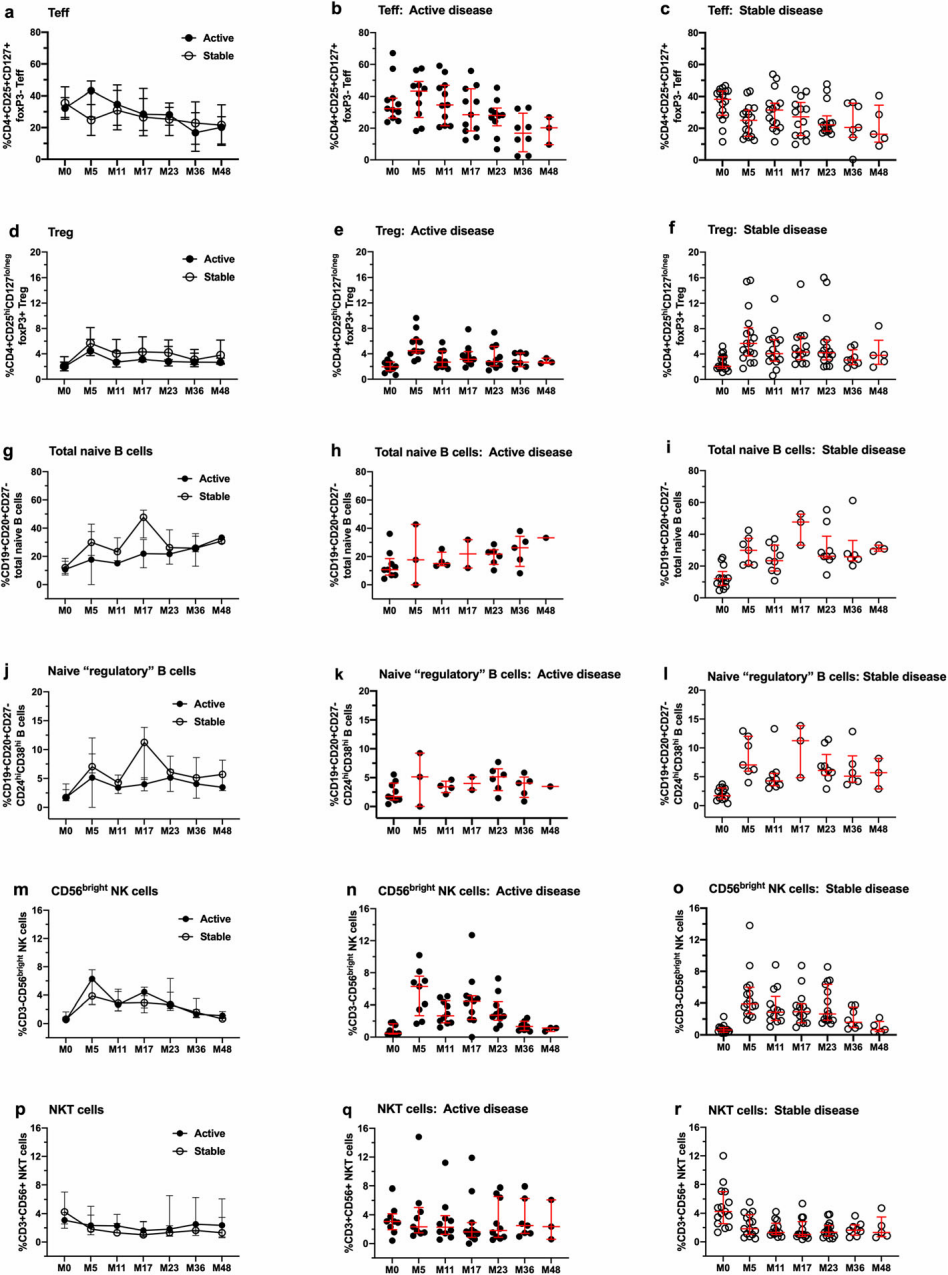
Lack of significant differences in selected lymphocyte subsets stratified for patients with active and stable disease activity during the 48-month study period. Each row represents data from one lymphocyte subset, with the left panels in each row illustrating between-subjects analyses, followed by within-subjects analyses for patients with active (middle panels) and stable (right panels) disease activity. **a**–**c** Changes in CD4+CD25+CD127+foxP3- Teff. **d**–**f** Changes in CD4+CD25^hi^CD127^lo/neg^ foxP3+ Tregs. **g**–**i** Changes in CD19+CD20+CD27- total naïve B cells. **j**–**l** Changes in CD19+CD20+CD27-CD24^hi^ CD38^hi^ B cells. **m**–**o** Changes in CD3-CD56^bright^ NK cells. **p**–**r** Changes in CD3+CD56+ NKT cells. Data represent median/IQR; closed symbols indicate patients with active disease, and open symbols indicate patients with stable disease. Data were subjected to between- and within-subject analyses using a linear mixed effects model for repeated measures

**Fig. 7:**
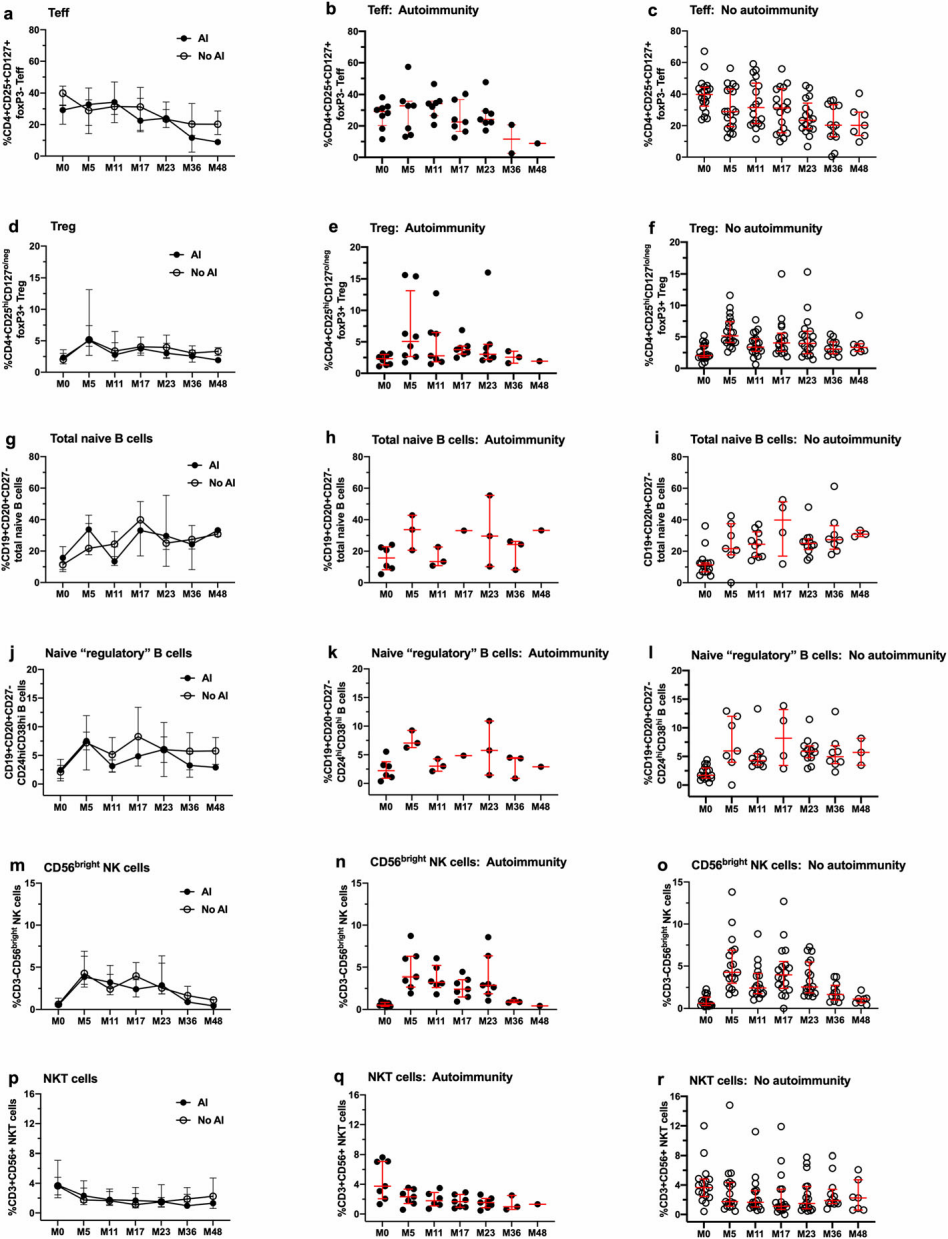
Lack of significant differences in selected lymphocyte subsets stratified for patients with and without secondary autoimmunity during the 48-month study period. Each row represents data from one lymphocyte subset, with the left panels in each row illustrating between-subjects analyses, followed by within-subjects analyses for patients with secondary autoimmunity (middle panels) and without secondary autoimmunity (right panels) disease activity. **a**–**c** Changes in CD4+CD25+CD127+foxP3- Teff. **d**–**f** Changes in CD4+CD25^hi^CD127^lo/neg^ foxP3+ Tregs. **g**–**i** Changes in CD19+CD20+CD27- total naïve B cells. **j**–**l** Changes in CD19+CD20+CD27-CD24^hi^ CD38^hi^ B cells. **m**–**o** Changes in CD3-CD56^bright^ NK cells. **p**–**r** Changes in CD3+CD56+ NKT cells. Data represent median/IQR; closed symbols indicate patients with secondary autoimmunity, and open symbols indicate patients without secondary autoimmunity. Differences over time between and within groups were not statistically significant (linear mixed effects model for repeated measures)

Analyses of correlations between immune measures and EDSS, number of new T2 lesions, T2 volume, number of new Gd+ lesions or BPF did not reveal statistically significant associations when assessed individually (data not shown). Lymphocyte subset and function data were stratified for the presence and absence of active disease (Fig. 6) or the presence and absence of secondary autoimmunity (Fig. 7). The data in both figures illustrate between group values (plots on the left), as well as individual values within each group (middle and right plots). No significant differences were observed over time for percentages of Teff (Figs. 6 and 7, panels a–c), Tregs (Figs. 6 and 7, d–f), total naïve B cells (Figs. 6 and 7, panels g–i), naïve “regulatory” CD24^hi^CD38^hi^ B cells (Figs. 6 and 7, j–l), NKT cells (Figs. 6 and 7, panels m–o) and CD3-CD56^bright^ NK cells (Figs. 6 and 7, panels p–r).

Although the changes in Teff did not differ between patients with active or stable disease, they showed different trends at M5, most clearly evident as an increase in patients with active disease (Fig. 6b) and a decrease in patients with stable disease (Fig. 6e). This trend held up for patients with and without relapses (supplementary Figure S6A-C), and less so for patients with and without Gd+ and T2 lesions, combined (supplementary Figure S6D-F).

There were no significant associations with disease status or secondary autoimmunity and Teff:Treg ratios, Treg function, Th1 and Th17 cells, CD3 + CD4+ and CD3 + CD8 + CXCR3+ and CCR5+ cells, naïve and memory CD4+ T cell subsets, total B cells, and total memory B cells (supplementary Figure S5).

Cell count data from clinical TBNK analyses, illustrated in supplementary Figure S1, revealed a typical temporal pattern of lymphopenia and repopulation in primary lymphocyte populations after each treatment course, consistent with published findings [23, 26–28, 56–59].

### Estimation of risk for lesion activity associated with immune measures

Exploratory analyses of potential associations between immune measures and the incidence of new T2 or Gd+ lesions at all timepoints were conducted using a random effect GEE Poisson model. The random effect estimates within-individual level associations between immune measures and risk of T2 or Gd+ lesion incidents. The overall fixed effect was the weighted average across multiple individuals. Since immune measures were standardized to a z score with a standard deviation of 1, the estimated rate ratio (rr) reflects a change in risk associated with immune measures that increase by one standard deviation from group means. In addition, because these analyses included measures at M0, they were conducted without consideration for a treatment effect. Low rate ratios (≤ 0.5, green/yellow), indicative of a reduced risk for Gd+ lesions, or a protective effect, were associated most strongly with regulatory cell subsets or functions, including Treg function and percentages of naïve “regulatory” B cells, CD3-56^bright^ NK cells, and Tregs (Table 2). High rate ratios (≥ 1.0, orange/red), indicative of increased risk for Gd+ lesions, were associated with general T cell populations, including proportions of CD3+, CD3+CD4+, CD3+CD8+, and total CD4+CD45RA+ naive T cells, as well as NKT cells, ratio of NKT:CD3-CD56^bright^ NK cells, and total CD19+CD20+CD27+ memory B cells. The data also reveal a high rate ratio for IL-2 (1.37, *p* = 0.02), a pro-inflammatory cytokine that promotes T cell activation and proliferation. Of note, an increase the percentage of CD4+CD25+CD127+foxP3- Teff by one standard deviation from the mean, with a rr of 0.93, was not associated with increased Gd+ lesion risk (*p* = 0.94; supplementary Table 1). Supplementary Table 1 shows additional phenotypes or functions that either did not yield rr values ≤ 0.5 or ≥ 1.0 or did not reach *p* values ≤ 0.05. 
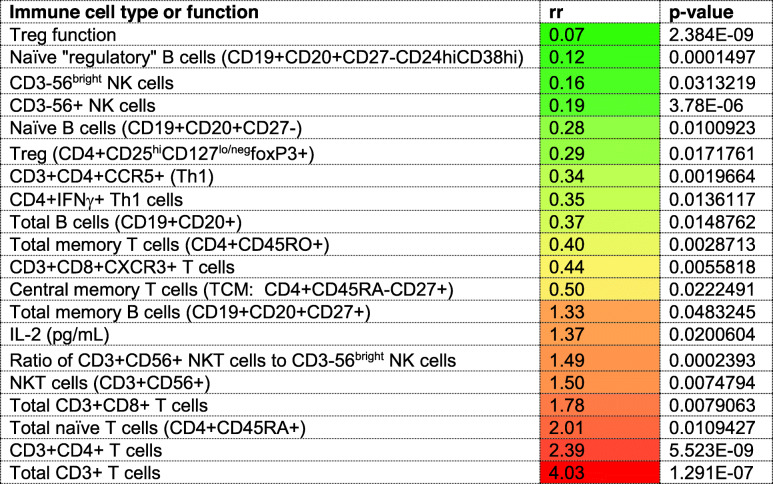

**Table 2:**
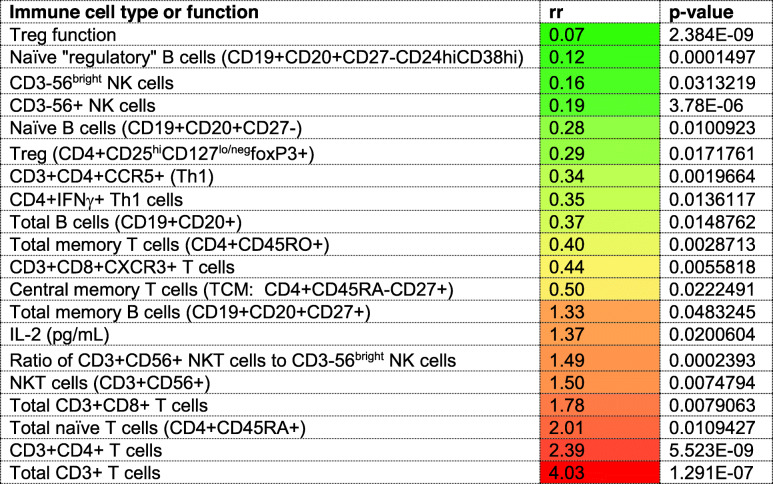
Within-individual associations between immune cell types or functions and risk of Gd+ lesions*

*Data represent findings from random GEE Poisson analyses sorted from low (green/yellow) to high (orange/red) rate ratios. Low rate ratios (≤ 0.5) indicate a protective effect, while rate ratios (≥ 1.0) indicate that the risk for Gd + lesions is associated with an increase in immune measures, represented by an increase by one standard deviation from the mean z score, as described in the methods section. Data are restricted to *p* values ≤ 0.05 after Benjamini-Hochberg corrections. All study timepoints were included in the analyses.

For new T2 lesions, CD3+CD8+CXCR3+ T cells were the only cell subset that showed significantly increased risk, with a rate ratio of 1.54 (*p* = 0.017; supplementary Table 1). The rate ratio for this subset showed the opposite trend for Gd+ lesions (rr = 0.44; *p* = 0.006; Table 2).

## Discussion

The data in this manuscript show that regulatory cell types that are enhanced and persist after alemtuzumab treatment include classical CD4+CD25^hi^CD127^lo/neg^foxP3+ Tregs, CD19+CD27-CD24^hi^CD38^hi^ naïve “regulatory” B cells, and CD56^bright^ NK cells. All were present at higher percentages within their respective parent populations after treatment, compared with baseline. Although similar findings have been reported for each of these subsets, very few studies have assessed all of them in the same patient cohort in long-term studies. In addition, the data expand our knowledge of the effect of alemtuzumab on the balance between regulatory and putatively pathogenic effector cell types, in which the ratios of CD4+CD25+CD127+foxP3- Teff:Treg, CD19+CD20+CD27+ memory B cells:naïve “regulatory” B cells, and CD3+CD56+NKT:CD56^bright^ NK cells were significantly reduced, in favor of regulation.

The Treg observations in this study are consistent with several reports that CD4+CD25^hi^CD127^lo/neg^ foxP3+ Tregs expand preferentially in the CD4+ T cell pool during the early stages of recovery, peaking at approximately 3–5 months and remaining elevated compared with baseline for approximately 2 years [23, 29, 30, 37]. Our findings expand these observations to include assessments of ratios between Teff:Treg, which were profoundly and persistently reduced over time. Teff:Treg ratios were similarly reduced when calculated on the basis of estimated Treg and Teff counts (data not shown).

The data provide both direct and indirect evidence of Treg competence. Direct evidence was provided in the form of significant rebound proliferation at M5 and M17 in PHA-activated PBMC depleted of Tregs, timepoints that follow cycles of alemtuzumab treatment at M0 and M12. Treg competence has been reported at 12 and 24 months post-treatment by De Mercanti and colleagues, who conducted enzyme-linked immunospot (ELISPOT) assays to detect rebound of myelin basic protein (MBP)-stimulated IFN-γ- and IL-17A-secreting cells in PBMC depleted of CD25+ cells [29]. In addition, Jones and colleagues employed classical Treg proliferation inhibition assays in alemtuzumab-treated patients to detect Treg competence at 36–48 months [37], but not earlier timepoints. Importantly, our data demonstrate that Tregs are not only functionally competent, but that they exhibit more robust competence at the early timepoints of M5 and M17.

Indirect evidence of Treg competence can be found in data showing that the majority of Tregs express CD39, an ectoenzyme associated with regulation of immune responses [60–62]. Our findings confirm reports of CD39+CD25^hi^ foxP3+ Tregs in the repopulating CD4+ T cell pool in alemtuzumab-treated patients [30, 37, 63]. The importance of CD39 expression in Tregs is supported by reports that the presence of CD39 stabilizes and promotes functional capacity in Tregs [64–67]. In addition, CD39+ Tregs have been reported to be reduced or functionally defective in MS patients [64, 68, 69], similar to original reports for classical Tregs [70, 71].

Although not directly assessed in this study, functionally competent regulatory B cells and CD56^bright^ NK cells may accompany the expansion of Tregs. Kim and colleagues reported that deficits in regulatory B cells, defined as CD19+CD24^hi^CD38^hi^ or CD19+PD-L1^hi^, are reversed up to 1 year following alemtuzumab treatment [22], and Grutzke and colleagues [10] have reported that CD19+CD27-CD38^hi^ B cells from MS patients secrete high levels of IL-10. In addition, Gross et al. reported that CD56^bright^ NK cells retain potentially regulatory cytolytic function at 6 months after alemtuzumab treatment [21]. Of special interest is that changes in phenotypically naïve “regulatory” B cells and CD56^bright^ NK cells were more robust, persisted for at least 2 years and exhibited less variability than those in Treg subsets, suggesting that they may play key roles in alemtuzumab’s durable mechanism of action.

Because regulatory populations in each compartment interact with each other to fine-tune regulation of immune responses, clinical efficacy in alemtuzumab-treated patients may also reflect the sum of a network of these interactions. For example, Tregs are capable of directly and indirectly suppressing B cells to control antibody production [72]. Regulatory B cell subsets not only inhibit Th1 and Th17 cells, but also convert CD4+ T cells into Tregs or IL-10 producing Tr1 cells via production of regulatory cytokines, including IL-10, IL-35, and/or transforming growth factor-beta (TGF-β) [73, 74]. In addition, subsets of NK cells promote Treg development [75–77], and regulatory T, B, and NK subsets all interact with antigen presenting cells to promote a tolerogenic cascade [70, 74, 78, 79]. Furthermore, several subsets of regulatory CD8+ T cells [80] are capable of contributing to a regulatory environment.

Our study included assessments of changes in T cells that express chemokine receptors, initially as a surrogate measure of Th1 cells, which express high levels of CXCR3+ and CCR5+, and Th2 cells, which are more likely to express CCR3 and CCR5. We originally hypothesized that an anti-inflammatory immune environment following alemtuzumab treatment may consist of increases in CCR3+ and/or CCR5+ Th2 cells and decreases in CXCR3+ and/or CCR5+ cells. Our findings do not support the use of chemokine receptor expression as indicators of Th1 or Th2 bias in CD4+ T cells, especially since cytokine secretion patterns, or additional functional assessments, were not included. However, the data clearly indicate that CD4+ and CD8+ T cells are activated and show enhanced capacity for migration in alemtuzumab-treated patients. Future studies might benefit from more detailed functional and phenotypic characterization of chemokine receptor bearing T cells.

A recent study published by Wiendl and colleagues reported that no clear immunological signals of efficacy or secondary autoimmunity could be identified in alemtuzumab-treated patients from the pivotal CARE-MS trials up to 24 months after treatment [81]. Their study assessed lymphocyte measures based on cell counts and included multiple phenotypes, many similar to those included in our study, as well as clinical measures that included relapses, 6-month confirmed disability worsening (CDW) based on EDSS and MRI activity, consisting of new Gd+ T1 lesions in current MRIs or new T2 hyperintense lesions since the last MRI. Our findings are generally consistent with this report.

However, patients in our cohort with stable disease showed a trend for reduced CD4+CD25+CD127+foxP3- Teff cells, most evident at M5, and Teff:Treg ratios that remained reduced from M5 to M48. In addition, at month 11, prior to the second course of alemtuzumab infusions, the Teff:Treg ratio appeared to be reverting to baseline in patients with active disease. This also occurred, to a lesser extent, in patients with and without relapses or Gd+ and T2 lesions. Future studies with larger numbers of patients are needed to determine if lack of depletion or reduced depletion of CD4+CD25+CD127+foxP3- Teff occurs in patients who exhibit disease activity on alemtuzumab, and as such, might serve as a potential biomarker for a suboptimal treatment response. To our knowledge, Teff defined as CD4+CD25+CD127+foxP3- cells have not been monitored in clinical studies, but we considered them as possible representatives of a general population of activated T cells that either exhibit effector functions or contain specific subsets of effector T cells. Thus, it would be of interest to determine if CD4+CD25+CD127+foxP3- Teff express markers or functions characteristic of pro-inflammatory “effector” T cells, such as IL-17A and IFN-γ.

Since MS is clearly an immunopathogenic disease, it is difficult to understand why analyses using linear mixed effects models for repeated measures have failed to more clearly identify immune markers of clinical status, especially since a large number of patients and immune measures were included in the study published by Wiendl and colleagues. We are either not on the right track with selection of immune measures for analyses, or we need to develop a different strategy of thinking about the cell types that may be involved. In view of the dynamic, plastic and heterogeneic nature of immune responses in general, and in immune repopulation in response to lymphopenia, it seems reasonable to consider relative contributions of multiple cell types. The results of the exploratory random generalized estimating (GEE) Poisson analyses of the risk for Gd+ and T2 lesions associated with increases in immune measures support this proposition and provide preliminary guidance for identification of relevant immune measures in future studies in larger patient cohorts. Of note, we found that the risk for Gd+ lesions (expressed as a rate ratio > 1.0), increased significantly when the percentage of general T cell populations (including CD3+ and CD4+ CD4+ T cells, CD4+CD45RA+ total naïve T cells), CD3+CD56+ NKT cells, and CD19+CD20+CD27+ memory B cells increased by one standard deviation at any timepoint. By contrast, an increase in regulatory cell types, including naïve “regulatory” CD24^hi^CD38^hi^ B cells, CD3-CD56^bright^ NK cell Tregs, and Treg function was associated with reduced risk for Gd+ lesions, or a protective effect (expressed as a rate ratio < 0.5). These findings support the possibility that potentially pathogenic “effector” subsets in the T cell, B cell, and NK cell compartments contribute to inflammatory lesion activity and that, as we have proposed, multiple regulatory T, B, and NK subsets and functions are capable of contributing to a protective effect.

In most patients, concentrations of IL-2 in supernatants from PHA-stimulated PBMC were dramatically reduced from M5 to M23, along with IFN-γ and IL-17A. Several investigators have assessed cytokine secretion patterns in serum and PBMC from alemtuzumab-treated patients [23, 29, 30, 37, 82], but not by CBA in supernatants from PHA-activated PBMC. Reductions in IL-2, IFN-γ, and IL-17A may reflect persistent depletion of total circulating CD4+ T cells, or, especially for IL-2, could be due to heightened susceptibility of repopulating T cells to activation-induced cell death following alemtuzumab treatment [83]. However, an increase in IL-2 concentrations was associated with increased risk for Gd+ lesions (rr = 1.33, *p* = 0.048) with possible implications for a less than adequate response to alemtuzumab. Interestingly, an increase in IL-10 concentrations yielded a rate ratio of 0.58 (*p* = 0.04) for Gd+ lesions, suggesting a possible protective effect.

CD3+CD8+CXCR3+ cells were the only cell type that showed a significant association with T2 lesions, which are generally a reflection of longer-term demyelination and axonal damage, and not active or acute inflammation. CD8+ T cells have long been known to outnumber CD4+ T cells in the CNS of MS patients and there is clear evidence of associations of CD8+ T cell subsets with demyelination and axonal damage [84–87]. Although total CD3+CD8+ T cells did not show a significant association with T2 lesions in the current study, the data suggest different mechanisms of immunopathogenesis for Gd+ and T2 lesions, and thus, support more thorough investigation of CD8+ T cells in the future.

Although we interpret the compelling preliminary findings from the exploratory random GEE Poisson analyses cautiously, they have identified several putative early immunologic biomarkers with potential to identify patients at risk for disease activity. It would be of great interest to apply this type of analyses to the substantially larger CARE-MS datasets, not only to confirm and validate the relative risk of lesion activity with pathogenic and regulatory lymphocyte subsets, but to determine if immunological signals of a treatment effect, or secondary autoimmunity can be identified.

At least 40% of patients treated with alemtuzumab develop secondary autoimmunity following alemtuzumab treatment and the incidence peaks approximately 3 years after the first treatment course [19]. This is not typically seen with other MS therapies that cause lymphopenia or specifically target B cells, with perhaps an exception for daclizumab, which targets NK cells [88]. We did not observe differences in T cell subsets or Treg function in patients with and without secondary autoimmunity, in agreement with others [37, 81]. It has been suggested that hyper-repopulation of B cells, especially naïve B cell subsets, in the absence of T cell regulation predisposes patients to secondary autoimmunity [26]. Although we also observed hyper-repopulation in total naïve B cells in our cohort, these cells also contained CD24^hi^CD38^hi^ naïve “regulatory” cells that showed similar kinetics of change, and analyses of changes in CD19+CD20+CD24^hi^CD38^hi^ cells in the absence of CD27 as a marker yielded similar patterns (data not shown). None of the additional B cell or NK measures that we assessed showed significant differences in percentage or kinetics in patients with and without secondary autoimmunity. Perhaps the best evidence of immunological mechanisms underlying secondary autoimmunity in alemtuzumab-treated patients is from two studies published by Jones and colleagues. The first revealed that serum IL-21 was at least 2-fold higher in a cohort of 32 patients who developed autoimmunity compared with 27 patients who did not, a phenomenon that may reflect a genetic predisposition [83]. Although IL-21 was not detected in supernatants from PHA-stimulated PBMC, we did not measure serum IL-21 in this study. The second study provides evidence that homeostatic proliferation of pro-inflammatory CD4+ and CD8+CCR7-RA- or CD45RA+ memory T cells with a restricted T cell repertoire drives the risk for secondary autoimmunity [37]. Although we assessed changes in naïve and memory CD4+ T cell subsets using CD27 instead of CCR7 as a marker, none of them revealed an association with presence of secondary autoimmunity. The identification of a practical immunological biomarker of the risk for secondary autoimmunity remains a high priority.

The repopulation of subsets occurred in relatively distinct temporal patterns, summarized in Table 3. Of special interest are changes that were detected at M5 and sustained throughout the remaining timepoints, or at least to M23, compared with baseline values, as they seem most likely to contribute to the clinical efficacy of alemtuzumab. In addition, changes in several subsets occurred in the majority, if not all patients, including reduced percentages and counts of CD3+ T cells and CD3 + CD4+ T cells, reductions in concentrations of IL-2 and IFN-γ in PHA-stimulated PBMC supernatants, reduced percentages of total memory B cells and increased proportions of naïve “regulatory” B cells.

**Table 3:** Summary of findings with potential to indicate mechanisms underlying clinical efficacy in alemtuzumab-treated patients

Measure	Change relative to M0
% regulatory T cells (Treg: CD4+CD25^hi^CD127^lo/neg^ foxP3+)	Sustained increase from M5 through M23 (Fig. 1)
% effector T cells (Teff: CD4+CD25+CD127+foxP3-)	No change from M0 (Fig. 1)
Ratio of Teff:Treg in PBMC	Sustained reduction from M0 through M23 (Fig. 1)
Regulatory T cell function	Increased at M5 and M17 (Fig. 1)
% memory B cells (CD19+CD27+)	Sustained reduction from M5 through M36 (Fig. 3)
% naïve “regulatory” B cells (CD19+CD20+CD27-CD24^hi^CD38^hi^)	Sustained increase from M5 through M23 (Fig. 3)
Ratio of memory B cells:naïve “regulatory” B cells	Sustained reduction from M5 through M23 (Fig. 3)
% NK cells (CD3-CD56+)	Sustained increase from M5 through M23 (Fig. 4)
% "regulatory" NK cells (CD3-CD56^bright^)	Sustained increase from M5 through M36 (Fig. 4)
Ratio of NKT cells:CD3-CD56^bright^	Sustained reduction from M5 through M36 (Fig. 4)
IL-2 (pg/mL)	Sustained reduction from M5 through M23 (Fig. S4)
IFN-γ (pg/mL)	Sustained reduction from M5 through M23 (Fig. S4)
IL-17A (pg/mL)	Sustained reduction at M5, M11 and M23 (Fig. S4)
IL-10 (pg/mL)	No change from M0 (Fig. S4)

There are several pitfalls in the current study. The number of M36 and M48 samples was low compared with M0–M23, limiting firm insights into more long-lasting clinically significant changes. Some measures may have been altered in samples shipped overnight, affecting comparisons with studies using fresh samples processed on the same day as collection. The number of samples available for B cell subset analyses was low compared with samples processed after overnight shipment, especially at M17, though the findings are consistent with observations published previously for B cell subsets in alemtuzumab-treated MS patients [22, 26, 31, 56]. Treg function was assessed in an unconventional assay involving rebound proliferation in PBMC depleted of Tregs, making it difficult to compare results with those in which classical proliferation or pro-inflammatory cytokine inhibition assays were employed. In addition, the identity of proliferating cells in CD25-depleted PBMC, and the possibility that depletion of CD25+CD8+ T cells, B cells, and NK cells might affect the assay were not assessed. The current study was designed to survey longitudinal changes in primary T cell, B cell, and NK cell subsets and did not include deep phenotyping and functional assessments in purified CD4+, CD19+, or CD56+ lymphocytes. This is important, since multiple subsets of regulatory CD4+ Tregs [70], CD8+ [80] regulatory B cells [74], and CD56^bright^ NK cells [89] with more specific and distinct surface phenotypes have been described, as is the case for naturally heterogenous effector lymphocyte subsets [90]. Finally, the study did not include healthy controls or untreated MS patients, which may have provided an additional perspective on the implications of the findings.

The efficacy of currently approved disease modifying treatments for MS is largely linked to the ability to deplete, suppress, or modify pathogenic T and B cells [2]. However, therapies that deplete both T and B cells also result in expansion or unmasking of regulatory cell types, most evident in treatment with cladribine [11], hematopoietic stem cell transplantation [91], and daclizumab [5]. A similar phenomenon occurs in alemtuzumab-treated renal transplant patients [92]. It is tempting to speculate that these findings support the possibility that regulation is a default state in the immune system that can be revealed during recovery from lymphopenia, and in part, recapitulates the developmental regulatory bias known to occur in the prenatal or neonatal periods (reviewed by Simon and colleagues, [93]).

## Conclusions

The data in this communication indicate that the immune environment in alemtuzumab-treated multiple sclerosis patients with relapsing-remitting disease is biased in favor of regulatory lymphocyte subsets that are not restricted to the T cell compartment, but also involve robust increases in the percentages of phenotypically regulatory B cell and NK cell subsets. Clinical efficacy may reflect the sum of interactions among these subsets to provide more effective control of multiple pro-inflammatory effector cell types. The data also indicate that CD4+ and CD8+ T cells that express chemokine receptors are enriched in the repopulating lymphocyte pool. Although clear immunological signals of efficacy or incidence of secondary autoimmunity were not identified, exploratory random GEE Poisson analyses support the possibility that the relative balance of several key potentially pathogenic and regulatory subsets is associated with the risk for or protection from inflammatory Gd+ lesions, while the risk for T2 lesions is associated with CD3+CD8+CXCR3+ T cells. Thus, the study identifies biomarkers with potential to predict future disease activity or stability. Future studies are needed to validate these findings and to refine our understanding of the mechanisms of action and risk for secondary autoimmunity in alemtuzumab-treated patients, as well as immunopathogenesis in MS.

## Supplementary information

**Additional file 1: Supplementary Figures.**

**Additional file 2: Table S1 and supplementary figure legends.** Increases in immune cell types or functions lacking statistically significant associations with risk of Gd+ and T2 lesions, with an exception for CD3+CD8+CXCR3+ T cells and T2 lesions (in bold type). **Figure S1.** Typical patterns of changes in lymphocyte and monocyte counts assessed every six months following alemtuzumab treatment in whole blood assessed in clinical TBNK assays. **Figure S2.** Expanded characteristics of Treg phenotypes in PBMC. **Figure S3.** Changes in naïve and memory CD4+ T cell subsets in whole blood following alemtuzumab treatment. **Figure S4.** Changes in cytokine secretion patterns in PBMC. **Figure S5.** Analyses of additional lymphocyte subsets stratified for active vs stable disease or presence and absence of secondary autoimmune disease. **Figure S6.** Lack of significant differences in percentages of CD4+CD25+CD127+foxP3- Teff cells stratified for patients with and without relapses (top three panels) or evidence of lesion activity on MRI (bottom three panels).

## Data Availability

Additional details on all methods will be shared with investigators upon request for purposes of replicating procedures and results. The data from this study will be provided by the corresponding author to qualified investigators upon reasonable request.

## Abbreviations

MS: Multiple sclerosis
RRMS: Relapsing-remitting multiple sclerosis
CD: Cluster of differentiation
FACS: Fluorescence-activated cell sorting
Treg: Regulatory T cells
Teff: Effector T cells
Th1: T helper 1 cells
Th2: T helper 2 cells
Th17: T helper 17 cells
NK: Natural killer cells
NKT: Natural killer T cells
IFN-γ: Interferon-gamma
IL: Interleukin
PHA: Phytohemagglutinin A
PBMC: Peripheral blood mononuclear cells
TBNK: T cell, B cell, NK cell (clinical laboratory phenotype assessment)
CBC: Complete blood count
FSC: Forward scatter (FACS)
SSC: Side scatter (FACS)
CBA: Cytometric bead array
rr: Rate ratio
