# Supplementary figures and images for: Repopulation of T, B, and NK cells following alemtuzumab treatment in relapsing-remitting multiple sclerosis
Source: J Neuroinflammation. 2020 Jun 15;17:189. doi: 10.1186/s12974-020-01847-9 (PMC7296935; doi:10.1186/s12974-020-01847-9)

## Slide 1
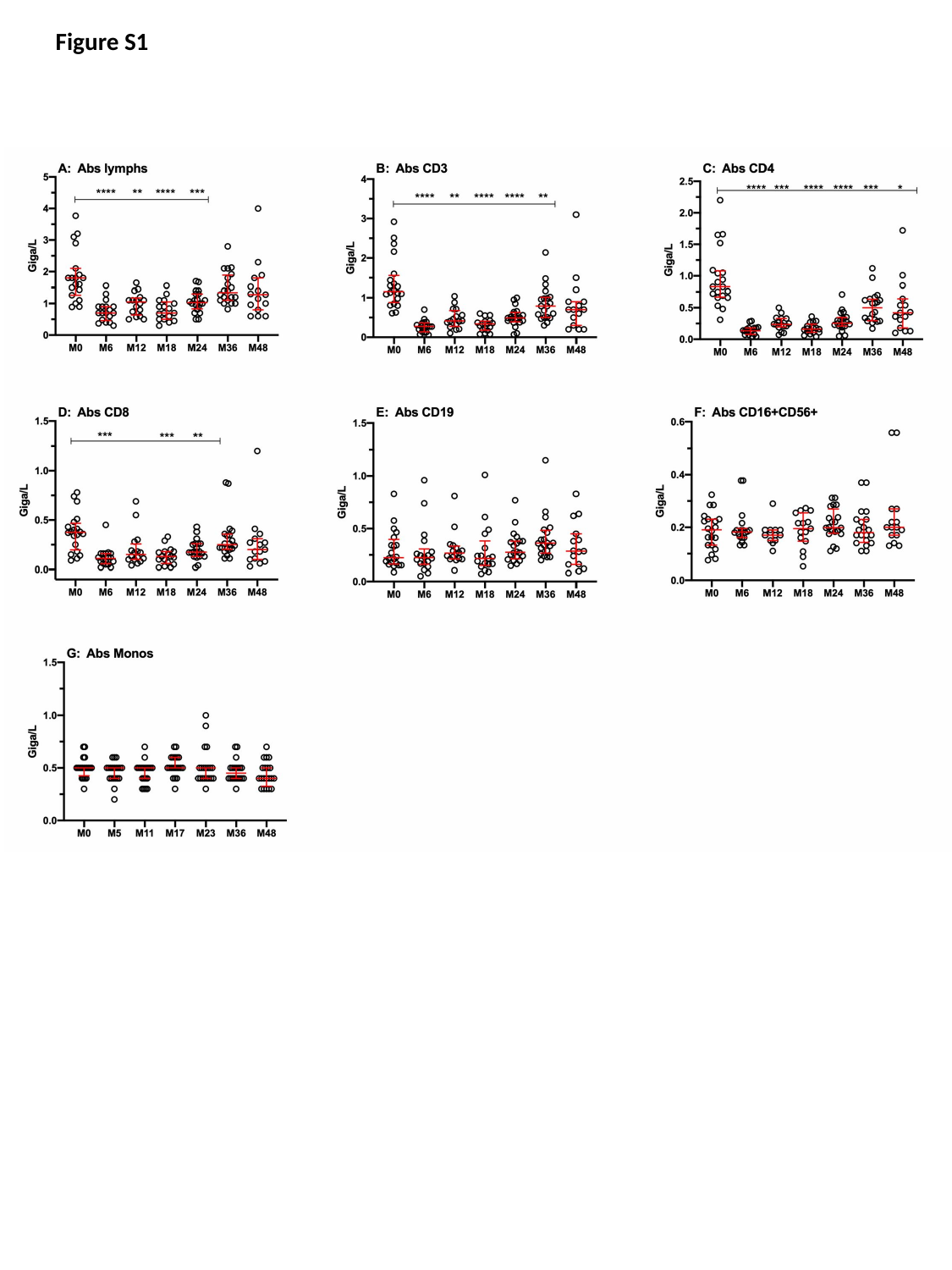

Figure S1

## Slide 2
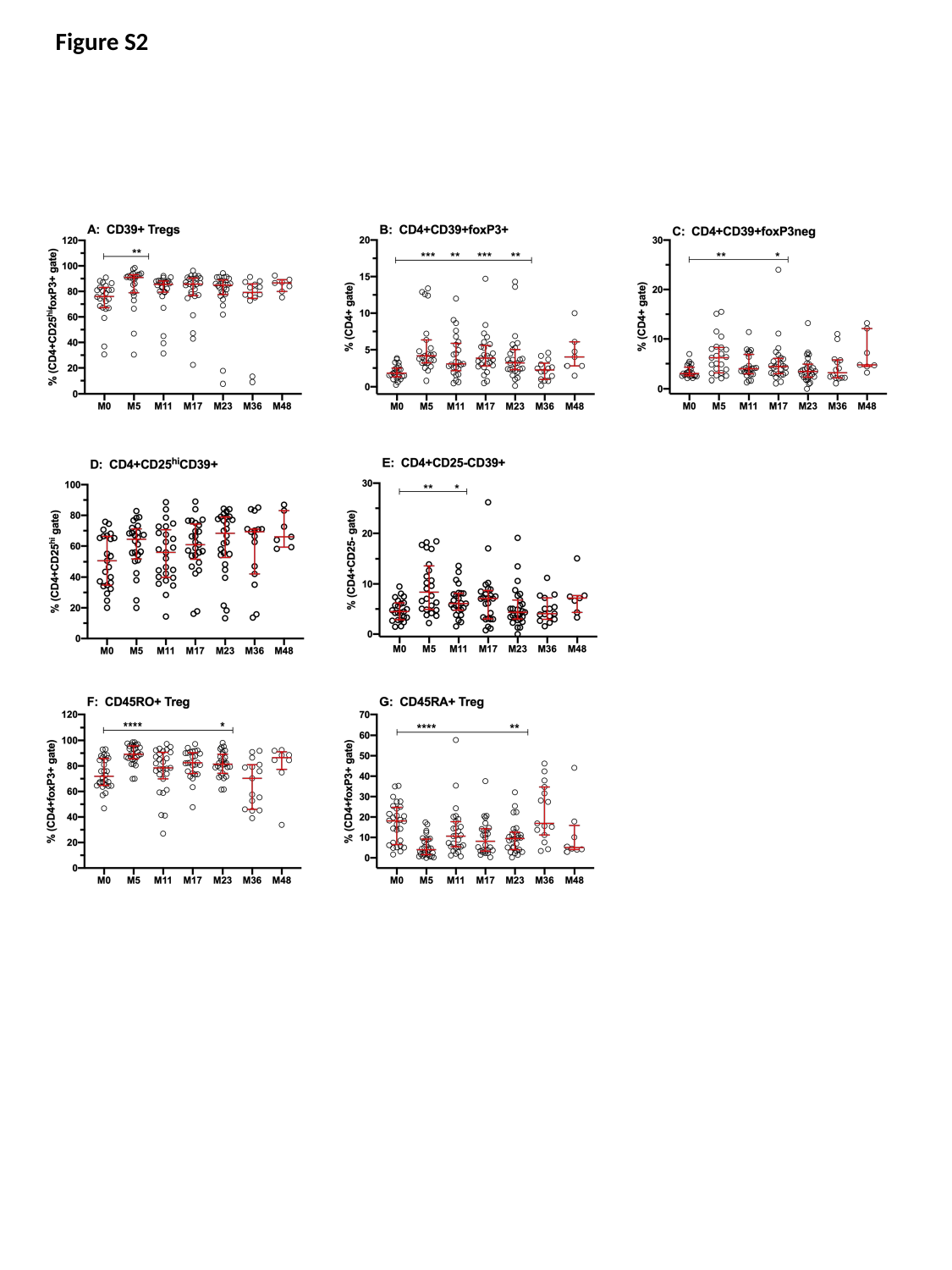

Figure S2

## Slide 3
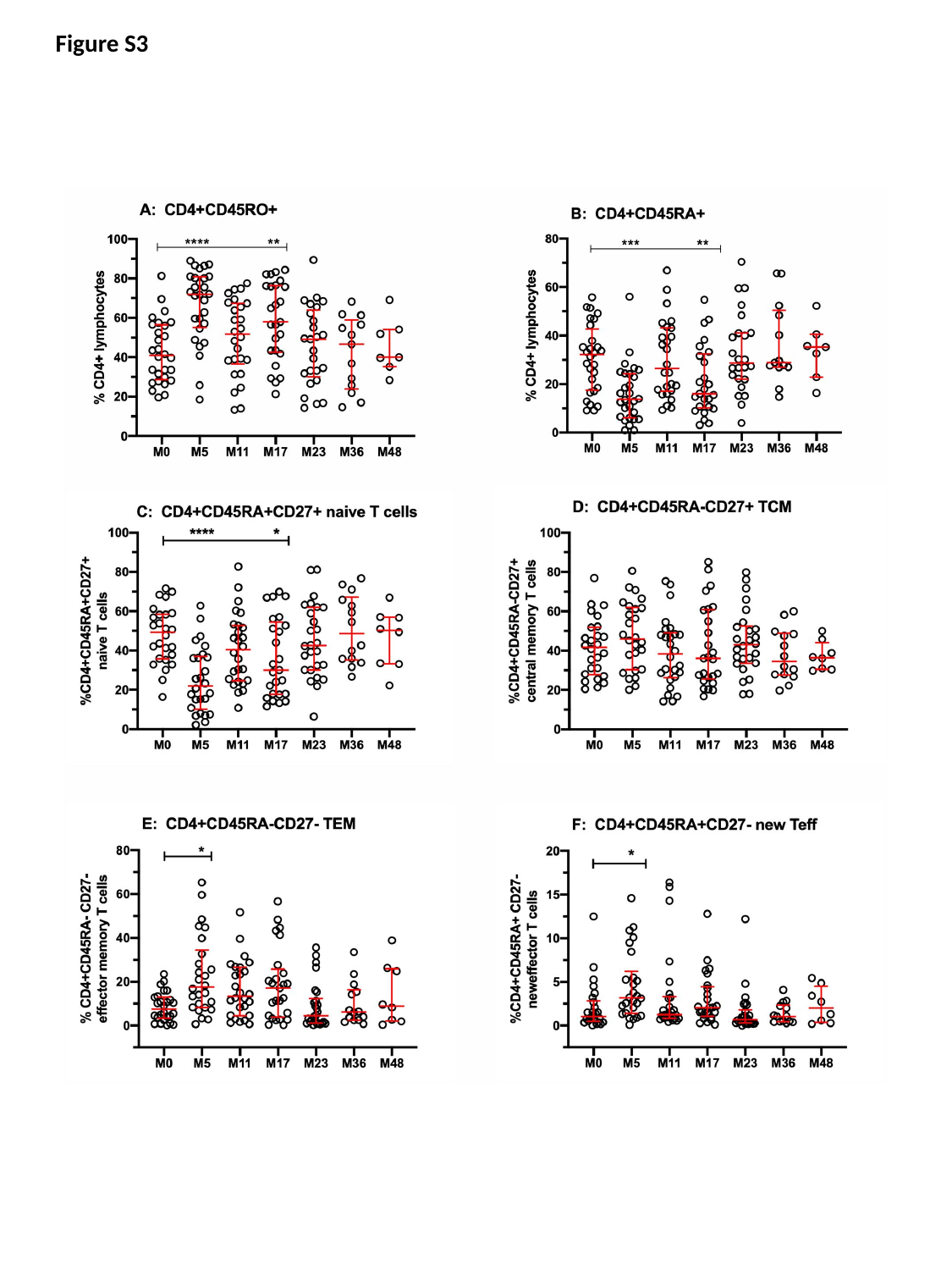

Figure S3

## Slide 4
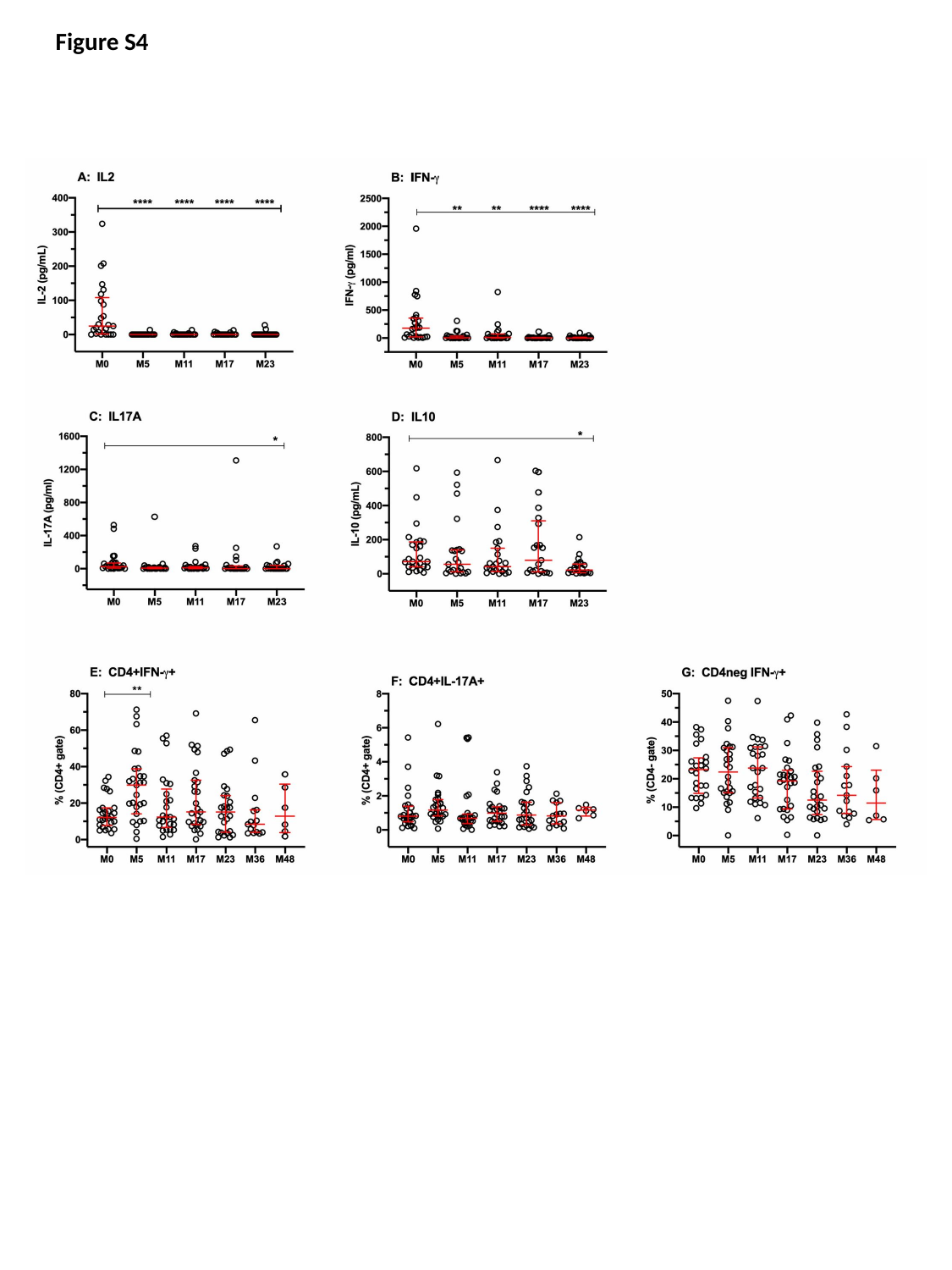

Figure S4

## Slide 5
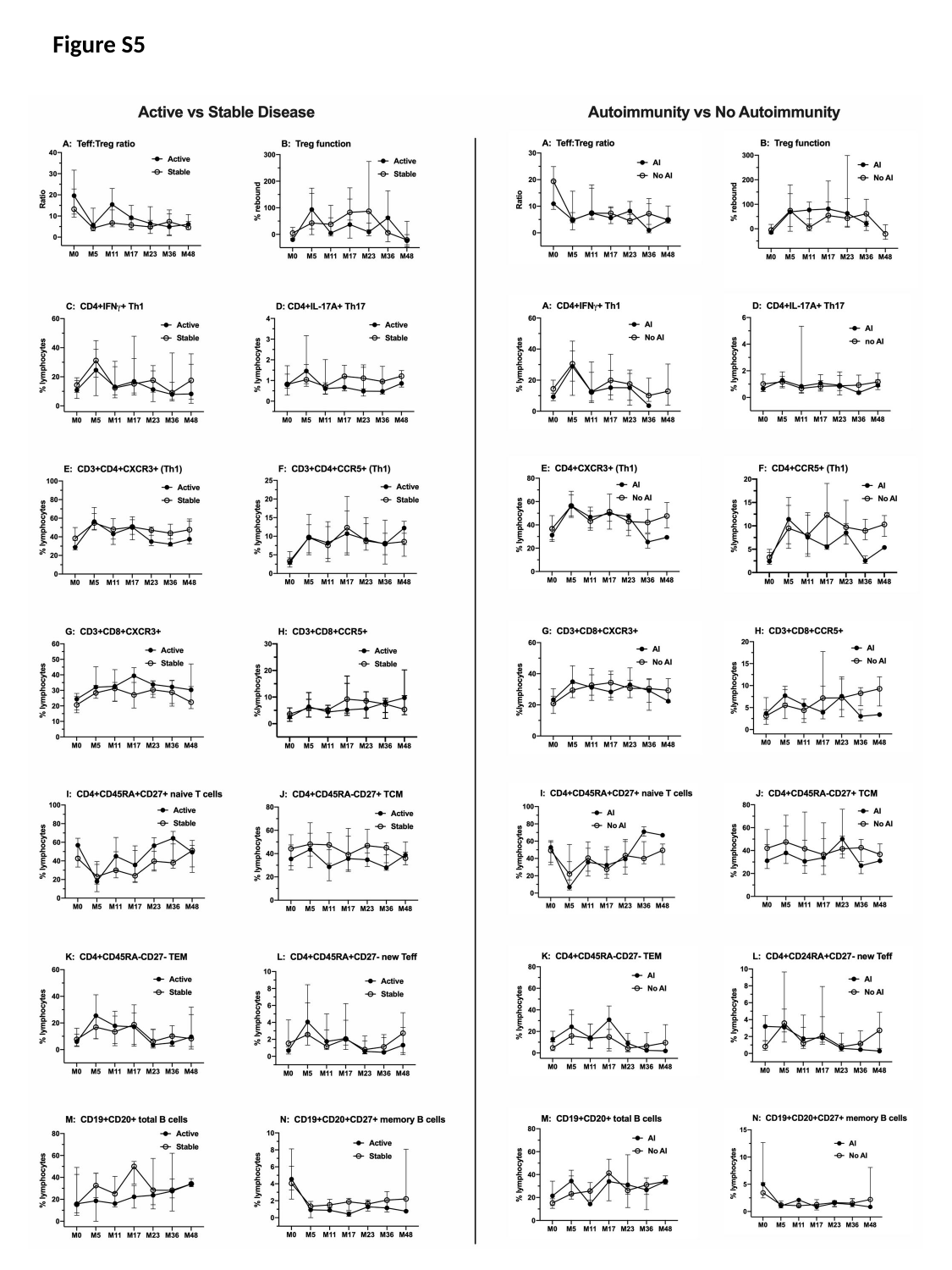

Figure S5

## Slide 6
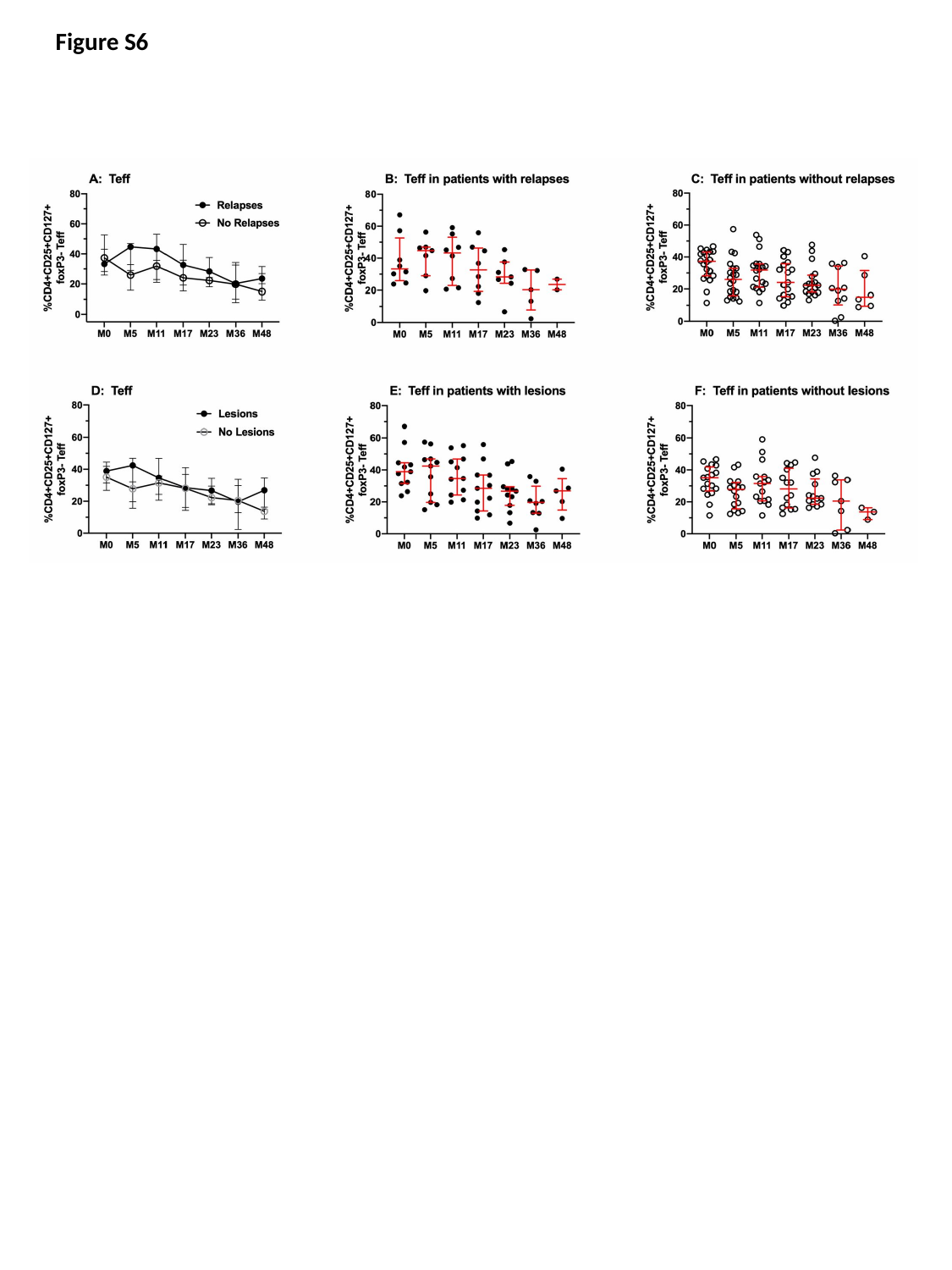

Figure S6

Supplement: Supplementary file 1 — Additional file 1: Supplementary Figures. [file 12974_2020_1847_MOESM1_ESM.pptx]
