# Supplementary material for: Repopulation of T, B, and NK cells following alemtuzumab treatment in relapsing-remitting multiple sclerosis
Source: J Neuroinflammation. 2020 Jun 15;17:189. doi: 10.1186/s12974-020-01847-9 (PMC7296935; doi:10.1186/s12974-020-01847-9)
Supplement: Supplementary file 2 — Additional file 2: Table S1 and supplementary figure legends. Increases in immune cell types or functions lacking statistically significant associations with risk of Gd+ and T2 lesions, with an exception for CD3+CD8+CXCR3+ T cells and T2 lesions (in bold type). Figure S1. Typical patterns of changes in lymphocyte and monocyte counts assessed every six months following alemtuzumab treatment in whole blood assessed in clinical TBNK assays. Figure S2. Expanded characteristics of Treg phenotypes in PBMC. Figure S3. Changes in naïve and memory CD4+ T cell subsets in whole blood following alemtuzumab treatment. Figure S4. Changes in cytokine secretion patterns in PBMC. Figure S5. Analyses of additional lymphocyte subsets stratified for active vs stable disease or presence and absence of secondary autoimmune disease. Figure S6. Lack of significant differences in percentages of CD4+CD25+CD127+foxP3- Teff cells stratified for patients with and without relapses (top three panels) or evidence of lesion activity on MRI (bottom three panels). [file 12974_2020_1847_MOESM2_ESM.docx]

**Supplementary Table 1: Increases in immune cell types or functions lacking statistically significant associations with risk of Gd+ and T2 lesions, with an exception for CD3+CD8+CXCR3+ T cells and T2 lesions (in bold type).**

| **Immune cell type or function** | **Lesion** | **rr*** | **p-value** |
| --- | --- | --- | --- |
| CD4+IL17A+ Th17 cells | Gd+ | 0.45 | 0.071383 |
| CD3+CD4+CXCR3+ (Th1) |  | 0.52 | 0.072915 |
| IL-10 (pg/mL) |  | 0.58 | 0.040365 |
| T effector memory T cells (TEM: CD4+CD45RA-CD27-) |  | 0.79 | 0.244907 |
| CD3+CD8+CCR5+ |  | 0.82 | 0.585868 |
| Teff (CD4+CD25+CD127+foxP3-) |  | 0.94 | 0.819047 |
| IL-17 (pg/mL) |  | 0.96 | 0.574791 |
| Ratio of Teff/Treg |  | 1.20 | 0.281685 |
| IFN-g (pg/mL) |  | 1.20 | 0.166076 |
| New Teff (CD4+CD45RA+CD27-) |  | 1.26 | 0.103185 |
|  |  |  |  |
| Total memory B cells (CD19+CD20+CD27+) | T2 | 0.32 | 0.161127 |
| IL-2 (pg/mL) |  | 0.45 | 0.729302 |
| Ratio of CD3+CD56+ NKT cells to CD3-56^bright^ NK cells |  | 0.46 | 0.067480 |
| Ratio of Teff/Treg |  | 0.71 | 0.199753 |
| CD3+CD4+ T cells |  | 0.71 | 0.127871 |
| Total CD3+ T cells |  | 0.74 | 0.201236 |
| IFN-g (pg/mL) |  | 0.82 | 0.835727 |
| IL-10 (pg/mL) |  | 0.82 | 0.282977 |
| NKT cells (CD3+CD56+) |  | 0.85 | 0.793409 |
| IL-17 (pg/mL) |  | 0.91 | 0.420195 |
| Central memory T cells (TCM: CD4+CD45RA-CD27+) |  | 0.92 | 0.820981 |
| Teff (CD4+CD25+CD127+foxP3-) |  | 0.93 | 0.933177 |
| Total naïve T cells (CD4+CD45RA+) |  | 0.94 | 0.900257 |
| New Teff (CD4+CD45RA+CD27-) |  | 0.96 | 0.964534 |
| CD3+CD8+CCR5+ |  | 0.98 | 0.975366 |
| Total B cells (CD19+CD20+) |  | 0.99 | 1.000000 |
| Total memory T cells (CD4+CD45RO+) |  | 0.99 | 1.000000 |
| Total CD3+CD8+ T cells |  | 1.00 | 0.987911 |
| CD3+CD4+CCR5+ (Th1) |  | 1.03 | 1.000000 |
| CD3+CD4+CXCR3+ (Th1) |  | 1.08 | 0.902724 |
| Total naïve B cells (CD19+CD20+CD27-) |  | 1.09 | 0.896128 |
| Treg function |  | 1.09 | 0.696094 |
| T effector memory T cells (CD4+CD45RA-CD27-) |  | 1.09 | 0.692561 |
| CD4+IFNg+ Th1 cells |  | 1.12 | 0.600649 |
| Treg (CD4+CD25^hi^CD127^lo/neg^foxP3+) |  | 1.15 | 0.263371 |
| CD3-56^bright^ NK cells |  | 1.20 | 0.271407 |
| Naïve "regulatory" B cells (CD19+CD20+CD27-CD24hiCD38hi) |  | 1.23 | 0.521709 |
| CD3-56+ NK cells |  | 1.23 | 0.288450 |
| CD4+IL17A+ Th17 cells |  | 1.30 | 0.171289 |
| **CD3+CD8+CXCR3+ T cells** |  | **1.54** | **0.016988** |

* Data represent findings from random GEE Poisson analyses sorted from low to high rate ratios (rr). If statistical significance were achieved, rr ≤ 0.5 would indicate that immune measures that increase one standard deviation from the mean z scores, as described in the methods, are associated with a protective effect, while rr ≥ 1.0 would indicate an increased risk for lesions. All study timepoints were included in the analyses.

**Figure S1**: **Typical patterns of changes in lymphocyte and monocyte counts assessed every six months following alemtuzumab treatment in whole blood assessed in clinical TBNK assays**. Numbers of lymphocytes (panel A) and CD3+ T cells (panel B) were significantly reduced from M6 to M24 and M6-M36, respectively, while CD4+ T cell counts (panel C) remained reduced throughout M48. CD8+ T cell counts (panel D) were reduced at M6, M18 and M24. By contrast, CD19+ B cell (panel E), CD16+CD56+ NK cell (panel F) and monocyte counts (panel G) did not show significant changes over time. Data represent median/IQR values for all patients at each timepoint; open circles indicate data points for each individual patient. P values indicate significant changes from baseline (M0) values as follows: ****p ≤ 0.0001, ***p ≤ 0.001, **p ≤ 0.01, * p ≤ 0.05; mixed effects ANOVA with Tukey’s corrections for multiple comparisons.

**Figure S2: Expanded characteristics of Treg phenotypes in PBMC.** The majority of CD4+CD25^hi^foxP3+ Tregs expressed CD39 and increased modestly, but significantly at M5 (panel A), though CD39 was expressed on both foxP3+ (panel B) and foxP3 negative (panel C) CD4+ T cells and showed a pattern of increase over time similar to classical Tregs (Figure 1, panel B). In addition, CD39 bearing Tregs were largely represented in CD4+CD25^hi^ gated cells (panel D), with little variability over time, while the percentage of CD39+ cells in the CD4+CD25- gate was increased at M5 and M11 (panel E). The majority of CD4+foxP3+ Tregs expressed the memory T cell marker CD45RO (panel F) and increased significantly at M5 and M23, while the percentage of CD45RA+ naïve T cells was reduced at M5 and M23 (panel G). Data represent median/IQR values for all patients at each timepoint; open circles indicate data points for each individual patient. P values indicate significant changes from baseline (M0) values as follows: ****p ≤ 0.0001, ***p ≤ 0.001, **p ≤ 0.01, * p ≤ 0.05; mixed effects ANOVA with Tukey’s corrections for multiple comparisons.

**Figure S3: Changes in naïve and memory CD4+ T cell subsets in whole blood following alemtuzumab treatment.** Panel A reveals significant sparing or expansion in the percentage of total CD4+CD45RO+ memory T cells at M5 and M17, and panel B shows reductions in CD4+CD45RA+ naïve T cells at the same time points. Panel C: CD4+CD27+CD45RA+ naïve T cells. Panel D: CD4+CD27+CD45RA- central memory T cells (TCM). Panel E: CD4+CD27-CD45RA- effector memory T cells (TEM). Panel F: CD4+CD27-CD45RA+ new effector T cells (new Teff). Data represent median/IQR values for all patients at each timepoint; open circles indicate data points for each individual patient. P values indicate significant changes from baseline (M0) values as follows: ****p ≤ 0.0001, ***p ≤ 0.001, **p ≤ 0.01, * p ≤ 0.05; mixed effects model ANOVA with Tukey’s corrections for multiple comparisons.

**Figure S4: Changes in cytokine secretion patterns in PBMC.** Panels A-D illustrate concentrations of cytokines measured in supernatants from PBMC collected at 48 hours following stimulation with PHA and assayed by CBA. IL-2 (panel A) and IFN-γ (panel B) were significantly reduced at all time points, while IL-17A was reduced at M5, M11 and M23, but not M17 (panel C). IL-10 secretion (panel D) remained unchanged over time. Data are expressed as median/IQR pg/mL. ****p ≤ 0.0001, ***p ≤ 0.001, **p ≤ 0.01, * p ≤ 0.05; compared with M0 using Kruskal-Wallis test. Panels E-G illustrate findings from PBMC subjected to overnight stimulation with PMA/calcium ionophore in the presence of brefeldin A, followed by intracellular staining for IFN-γ and IL-17A to identify percentages of Th1 and Th17 cells, respectively. For these analyses, viable lymphocytes were gated on CD4+ (panels E & F) or CD4 negative (panel G) cells. The percentage of CD4+IFN-γ+ Th1 cells increased significantly at M0 (panel E), with no change at remaining time points, while the percentage of CD4+IL-17A+ Th17 cells was low (panel F) and showed no clear trends for reduction over time. The percentage of IFN-γ producing cells in CD4 negative PBMC remained unchanged at all time points (panel G). Data represent median/IQR for all patients at each timepoint; open circles indicate data points for each individual patient. P values indicate significant changes from baseline (M0) values as follows: ****p ≤ 0.0001, ***p ≤ 0.001, **p ≤ 0.01, * p ≤ 0.05; mixed effects ANOVA with Tukey’s corrections for multiple comparisons.

**Figure S5: Analyses of additional lymphocyte subsets stratified for active vs stable disease or presence and absence of secondary autoimmune disease.** Data represent median/IQR values; closed symbols indicate patients with active disease or presence of secondary autoimmunity, open symbols indicate patients with stable disease or without secondary autoimmunity. Differences over time between subjects were not statistically significant (linear mixed effects model for repeated measures).

**Figure S6**: **Lack of significant differences in percentages of CD4+CD25+CD127+foxP3- Teff cells stratified for patients with and without relapses (top three panels) or evidence of lesion activity on MRI (bottom three panels).** The left panels (A&D) in each row illustrate between subjects comparisons, while the middle panels illustrate individual data for patients with relapses (panel B) or lesions (panel E). The right panels show individual data for patients with no relapses (panel C) or lesions (panel F). There was a trend for higher Teff in patients with relapses and lesions, but between and within group analyses did not reveal statistically significant differences (linear effects model for repeated measures).
